# Immunostimulatory effects of *Streptococcus sanguinis* extracellular membrane vesicles protect oral gingival epithelial cells from periodontal pathobiont damage

**DOI:** 10.1128/iai.00535-24

**Published:** 2025-02-19

**Authors:** Emily Helliwell, Isabella Rauch, Tim Nice, Justin Merritt, Jens Kreth

**Affiliations:** 1 Division of Biomaterial and Biomedical Sciences, School of Dentistry, Oregon Health & Science University 212162 , Portland, Oregon, USA; 2 Department of Molecular Microbiology and Immunology, Oregon Health & Science University 547642 , Portland, Oregon, USA; University of Illinois Chicago, Chicago, Illinois, USA

**Keywords:** *Streptococcus sanguinis*, commensals, oral biofilm, vesicles, immune response

## Abstract

The commensal *Streptococcus sanguinis* is highly prevalent in the oral cavity and characterized for its ability to inhibit growth of oral pathogens. Like many other cell types, streptococci produce extracellular membrane vesicles (EMVs), which contain specific molecular cargo and facilitate interactions with host cells. We previously demonstrated that EMVs from *S. sanguinis* are internalized by gingival epithelial cells (GECs) without causing cell death. Our aim is to characterize the effects of vesicles on eukaryotic cells. Microscopy studies of gingival epithelial cells inoculated with EMVs from wild-type and specific deletion mutants show differential uptake, with decreased uptake of ΔSSA1099 EMVs and increased uptake of ΔSSA1882 EMVs relative to SK36 EMVs. However, EMVs from wild-type and deletion mutants showed similar patterns in cytokine and chemokine secretion. Transcriptomic analysis of gingival epithelial cells inoculated with SK36 EMVs showed a downregulation of genes implicated in apoptosis as well as interferon signaling, while showing an upregulation of genes involved in cytokine production. Gelatin zymography results show that SK36 EMVs have a contrasting result on production of MMP2/9; MMP2 production is decreased while MMP9 is increased by 48 hours post-inoculation (hpi). Dual-inoculation studies demonstrate that prior internalization of *S. sanguinis* EMVs protects gingival epithelial cells from exposure to pathobiont *Porphyromonas gingivalis* outer membrane vesicles (OMVs), preventing dissociation and cell death. Our overall findings suggest that *S. sanguinis* EMVs trigger an immune response on gingival epithelial cells; however, this response suggests inhibition of some immune signaling pathways. Our results highlight an important role in commensalism, in which a microbe induces an immune response but avoids damage to host cells, thus discouraging infection by pathobionts.

## INTRODUCTION

The oral cavity is home to a diverse set of microorganisms, collectively shown to have a significant impact on overall oral health (1). Pathobionts such as *Porphyromonas gingivalis* or *Treponema denticola* within the subgingival space can drive the development of periodontal disease through the effects of specific virulence factors, causing a runaway, destructive inflammatory response, which leads to tissue degradation, dental bone resorption, and eventual tooth loss (2). In contrast, the oral cavity is also home to many commensal microbes. Oral commensals play various roles that promote microbe–host homeostasis, competitive inhibition, and/or neutralization of pathogenic microbes, or elicit host immune responses to inhibit colonization by pathogenic microbes (3, 4).

*Streptococcus sanguinis* is a gram-positive, facultative anaerobe and an early colonizer of the dental biofilm due to its ability to bind to sialylated glycoproteins (mucins) in the acquired pellicle that covers the tooth enamel surface (5). As an oral commensal, *S. sanguinis* closely interacts and coaggregates with other oral commensals like *Corynebacterium durum* and various other oral streptococci (6, 7). A key commensal role *of S. sanguinis* is to produce H_2_O_2_, which potently suppresses the growth of both caries and periodontal pathobionts (8). Paradoxically, S. *sanguinis* also elicits innate immune responses from gingival epithelial cells at levels similar to or exceeding that of major inflammophilic pathobionts like *Filifactor alocis* and *P. gingivalis* (4, 9). *S. sanguinis* produces an abundance of extracellular membrane vesicles (EMVs) as well, and these are similarly proinflammatory (9). EMVs are nanoparticles produced by both prokaryotic and eukaryotic cells that measure between 20 and 400 nm in diameter. Bacterial EMVs have been demonstrated to perform a myriad of functions, affecting quorum sensing, biofilm formation, dissemination, host cell signaling, etc. (10). EMVs are typically loaded with molecular cargo, including a diverse array of proteins, fatty acids, and nucleic acids that can directly affect the surrounding community and environment (10). In many cases, EMVs have been demonstrated to enter host cells, resulting in changes in immune signaling, secretion of proinflammatory cytokines and chemokines, as well as host cell reprogramming and associated systemic effects. EMVs from gut microbes play active roles in epithelial cell interactions and barrier integrity (11); however, far less is known about the impact of EMVs produced by the oral microbiome. Recently, we characterized the composition of *S. sanguinis* EMV cargo and revealed the growth conditions stimulating their production (9). *S. sanguinis* preferentially produces EMVs in media supplemented with glucose, and the EMVs are internalized into gingival epithelial cells within 24 h of inoculation. Analysis of the proteomic cargo revealed several proteins homologous to virulence factors produced by other streptococci, such as SSA2004 (12) and SSA1099 (13). In oral keratinocytes, *S. sanguinis* EMVs triggered a rapid induction of *IL6* and *CXCL8* gene expression, leading to increased production of IL6 and IL8. Based upon these results, we were interested to further characterize the effects of *S. sanguinis* EMVs on gingival epithelial cells in the current study, as well as determine the differences in host cell responses to an oral commensal vs a pathogen/pathobiont.

## RESULTS

### Characterization of EMV production in *S. sanguinis* deletion mutants

In our previous study (9), we demonstrated that *S. sanguinis* SK36 preferentially generated vesicles in media supplemented with glucose (over sucrose). Nanoparticle tracking analysis (NTA) showed a consistent yield of ~5 × 10^11^ particles per 1 L of media. A series of mutant strains were created by deleting proteins that have been identified as cargo in EMV through mass spectrometry analysis (Table 1) to further explore a potential role in EMV biogenesis or function. The criteria for choosing candidates for mutagenesis are as follows: (i) proteins must be present in at least three out of four prepared samples, (ii) proteins must not be critical for normal growth of *S. sanguinis*, and (iii) proteins are characterized as potential virulence factors in either *S. sanguinis* or other streptococci. Three candidates were identified. SSA1099 is annotated as an RTX adhesin and a calcium-binding hemolysin-like protein. It is a virulence factor that was first identified in *S. sanguinis* and was found to play a role in aggregation of platelets, thus contributing to infective endocarditis (13). SSA1882 is a member of the S8 subtilisin-like protease family with similarity to *Streptococcus thermophilus* PrtS (14). We previously demonstrated SSA1882 to be highly abundant in *S. sanguinis* EMVs (9). A PrtS ortholog (ScpA) of *Streptococcus pyogenes* serves as a virulence factor due to its cleavage of complement factors C5a and C3a, thereby limiting inflammatory activation (15, 16). SSA2004, annotated as *zmpB* (zinc metalloprotease B), is a member of the zinc metalloprotease family and is found in many other members of the *Streptococcus* genus. In *Streptococcus pneumoniae*, it is demonstrated to increase inflammation by inducing TNF*α* in the respiratory tract (12).

**TABLE 1:** SK36 mutants

Ensembl gene ID	COG	No. of samples	No. of peptides	Description	Gene symbol	Predicted localization	VFDB	VirulentPred
SSA_1882	R	4	9	C5a peptidase (prtS)	prtS	Membrane/extracellular	Yes	Virulent
SSA_1099	Q	4	4	Calcium-binding hemolysin-like protein, GN = SSA_1099	SSA_1099	Unknown	No	Virulent
SSA_2004	O	3	1	Zinc metalloprotease zmpB	zmpB	Membrane/extracellular	Yes	Virulent

**Fig 1:**
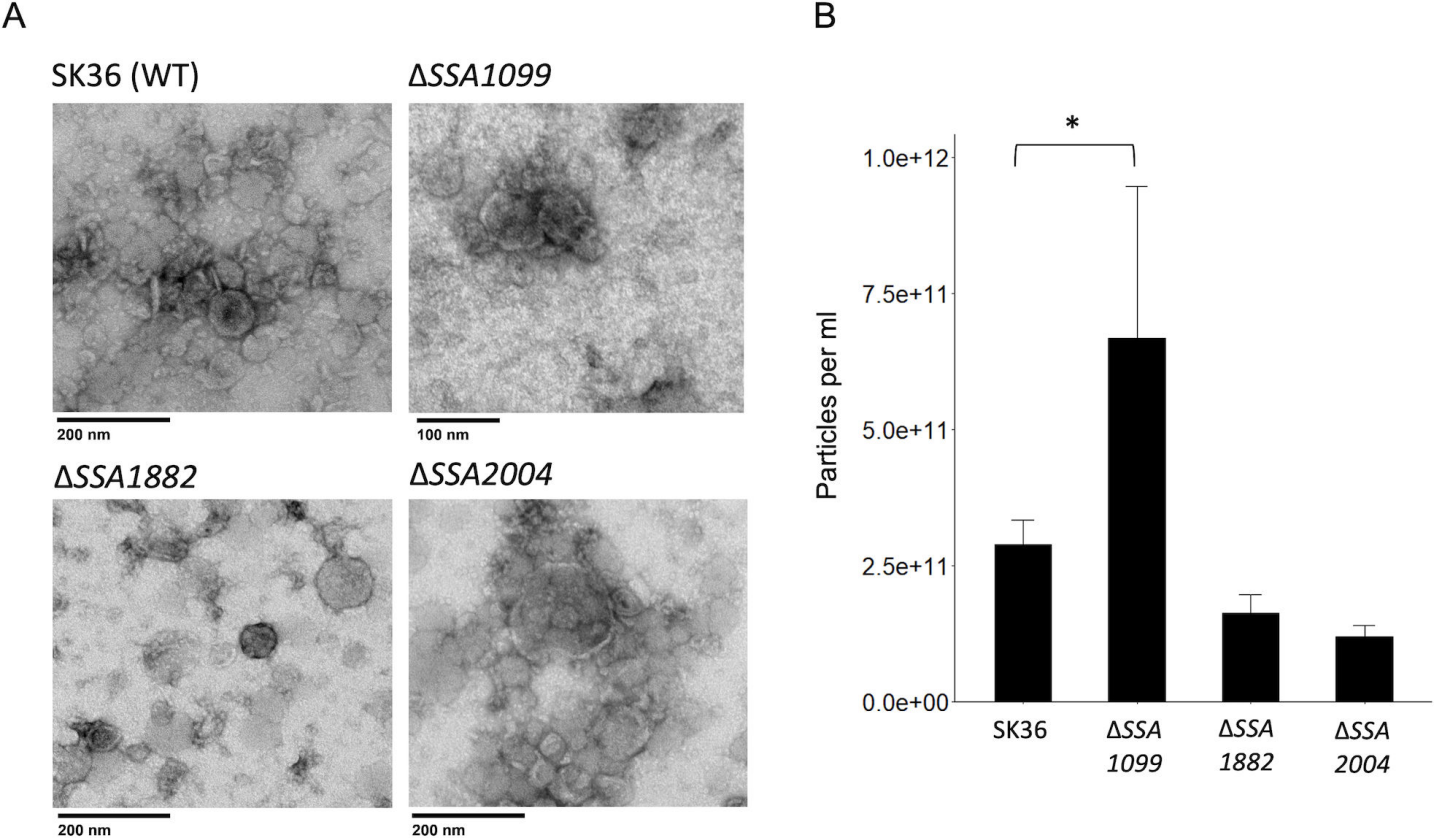
Characterization of EMVs produced by *S. sanguinis* WT and deletion mutants. (**A**) EMV counts by nanoparticle tracking analysis (NTA) comparing the number of particles per milliliter produced from 500 mL cultures of CDM + 3.4% glucose, incubated for 18 h at 5% CO_2_. Brackets with asterisks designate significant differences at *P* < 0.05. (**B**) Representative TEM images of EMVs isolated from wild-type and mutant *S. sanguinis* strains.

*S. sanguinis* deletion mutants of SSA1099 produce significantly higher amounts of vesicles compared to the wild-type (WT) SK36, while deletions of SSA1882 and *zmpB* (SSA2004) did not significantly impact EMV production (Fig. 1A). Transmission electron microscopy (TEM) images of EMVs from all deletion mutant strains showed no noticeable differences from *S. sanguinis* wild-type EMVs. (Fig. 1B). Likewise, distribution size analysis of the vesicles produced by the wild-type and all deletion mutants did not show any differences (Fig. S1).

### EMVs from ΔSSA1882 and ΔSSA1099 deletion mutants show differences in uptake by gingival epithelial cells

**Fig 2:**
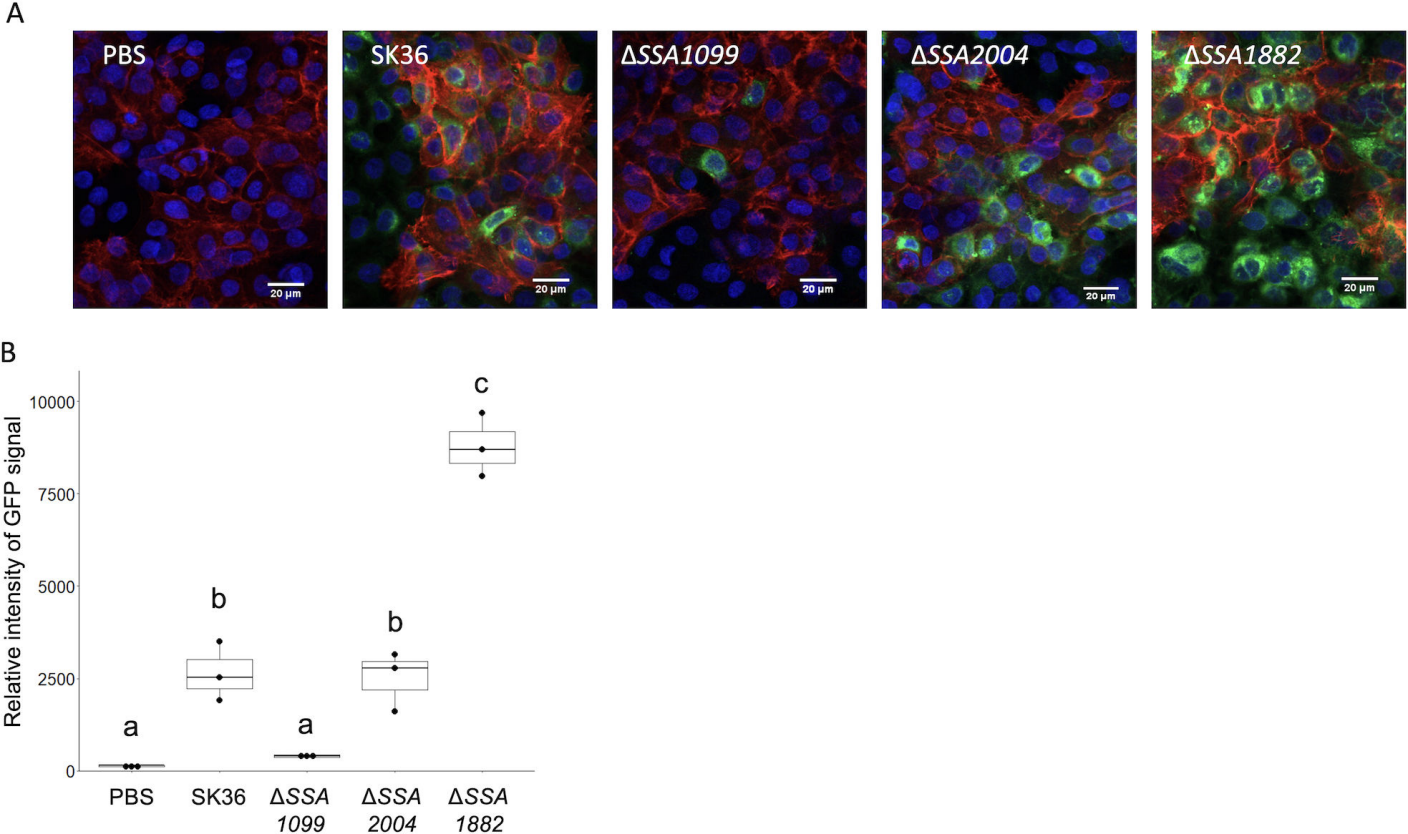
EMVs from SK36 and deletion mutants show differential internalization into gingival epithelial cells. (**A**) Representative images of gingival epithelial cells inoculated with PBS control, or 10^11^ EMVs from SK36 WT, ΔSSA1099, ΔSSA2004, and ΔSSA1882. GEC nuclei are stained with DAPI, Actin with Texas Red Phalloidin, and EMVs with DiO. (**B**) Boxplot showing green fluorescence intensity from two images per replicate, three replicates total in arbitrary units, calculated from Fiji (ImageJ). Significance was calculated using an ANOVA, with significance groups assigned by post hoc Tukey analysis at threshold *P* < 0.05.

Bacterial outer membrane vesicles (OMVs) and EMVs, including from *S. sanguinis*, can enter eukaryotic cells (17). For each of the strains, we tested EMVs stained with the lipophilic dye DiO for their abilities to enter the gingival epithelial cell line hTERT-TIGK CRL3397 (henceforth referred to as TIGK) within 24 h of inoculation. Uninternalized vesicles were removed from the cells prior to fixation and imaging. Representative photos are shown in Figure 2A. Z-stack images of inoculated TIGK cells to show EMV internalization can be viewed in Figure S2. We observed a similar pattern of staining in TIGK cells inoculated with SK36 EMVs as previously reported (9). However, cells inoculated with EMVs from the ΔSSA1882 mutant exhibited a brighter, more widespread DiO fluorescence. In contrast, cells inoculated with EMVs from the ΔSSA1099 mutant exhibited very little DiO fluorescence. EMVs from the Δ*zmpB* mutant appeared similar to that of SK36 EMVs. Fiji (ImageJ) software was used to gather semi-quantitative data on staining intensities (Fig. 2B). Staining intensities between both the PBS control and SK36, as well as the SK36 and ΔSSA1882 EMVs, were significantly different. In contrast, there was no significant difference between the PBS control and ΔSSA1099 EMVs. These results demonstrate that EMVs produced by different *S. sanguinis* deletion mutants are internalized into gingival epithelial cells at differing quantities, suggesting a direct or indirect role of these specific proteins in the internalization process.

### *S. sanguinis* EMVs induce cytokine production from GECs

**Fig 3:**
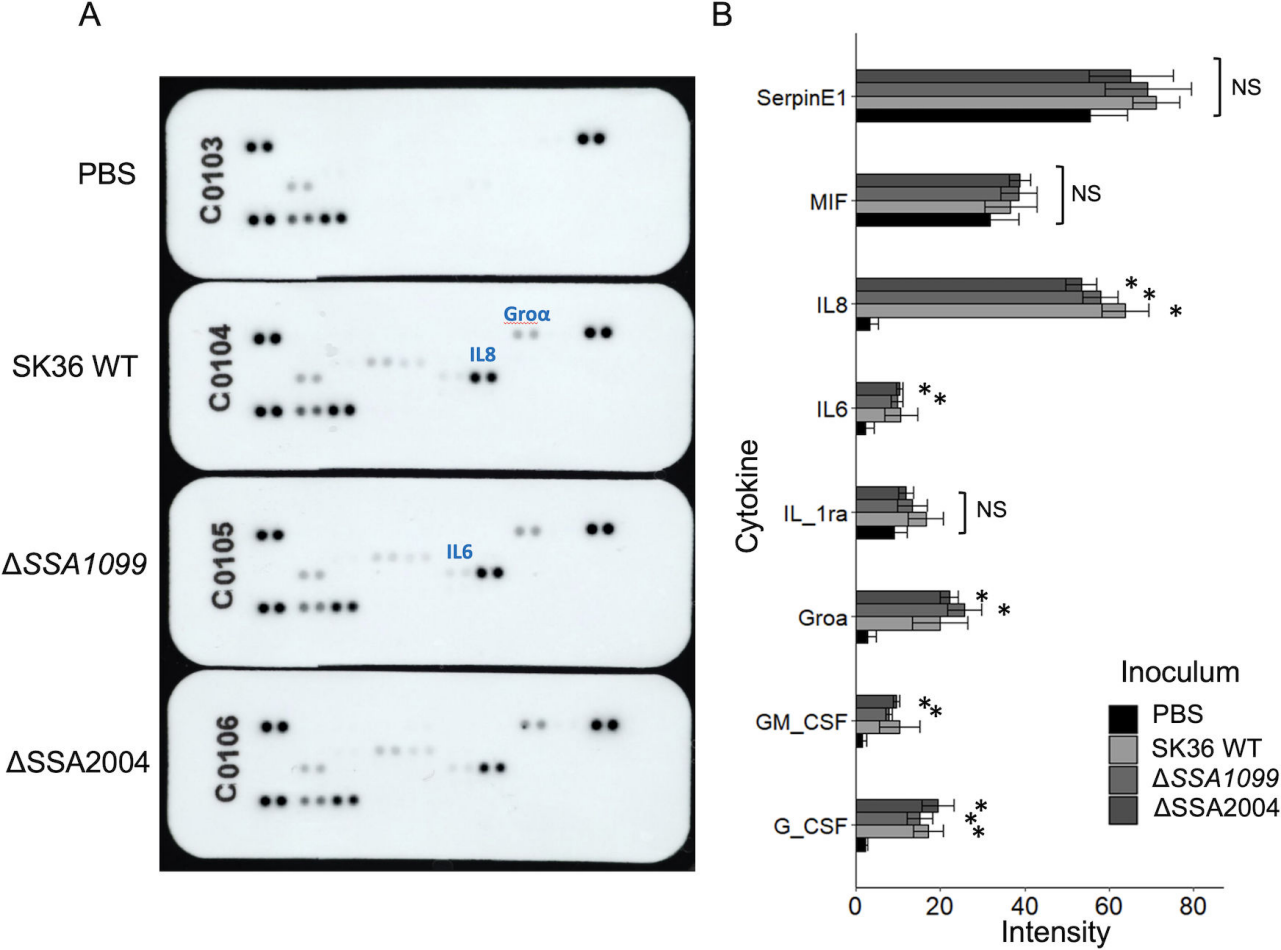
Quantitative comparison of cytokines released by EMV-inoculated TIGK cells. (**A**) Comparison of arrays exposed to supernatants from TIGK cells 24 h post-inoculation with either PBS, or 10^11^ EMVs from wild-type SK36, ΔSSA1099, or ΔSSA2004. Images show representative arrays from three biological replicates. (**B**) Comparison of mean intensity scores for cytokines secreted from TIGK cells, quantified by ImageJ. Bars represent the average of three biological replicates; * indicates a significant difference from PBS-inoculated control, NS designates no significant differences from PBS-inoculated control as calculated by an ANOVA.

Previously, we showed that inoculation of TIGK gingival epithelial cells with SK36 EMVs resulted in the rapid induction (6 hours post-inoculation [hpi]) of genes encoding selected cytokines (9). Here, we further characterized the cytokine profiles elicited by EMVs collected from the *S. sanguinis* mutant strains with deletions of genes predicted to be virulence factors in other streptococci, ΔSSA1099 and ΔSSA2004 at 24 hpi, to determine whether these mutations might affect the host response to the respective EMVs. Among the assayed cytokines, Serpin E1, MIF, and IL1ra showed no differences between the PBS-inoculated and EMV-inoculated GECs. In contrast, IL8 (interleukin-8) showed a significant increase between PBS- and EMV-inoculated GECs, with a 17.7-fold increase. However, this effect was not impacted in either *S. sanguinis* deletion mutants (Fig. 3A and B). This pattern was also seen with G-CSF (Granulocyte colony stimulating factor), which showed a 7.3-fold increase between PBS-inoculated and EMV-inoculated GECs. There were three quantifiable cytokines that showed no significant differences between PBS-inoculated and WT EMV-inoculated GECs; however, there was a significant difference between PBS-inoculated and deletion mutant EMV-inoculated cells. These were interleukin-6 (average 4.3-fold higher), Gro-*alpha* (average 8.3-fold higher), and GM-CSF (average 5.7-fold higher). Despite the differences in statistical significance among the EMVs, the general increase in cytokine production after EMV inoculation suggests that the deleted proteins likely do not play a role in cytokine secretion triggered by *S. sanguinis* EMV.

### RNA sequencing shows transcriptomic changes 24 h post-inoculation with *S. sanguinis* EMVs

**Fig 4:**
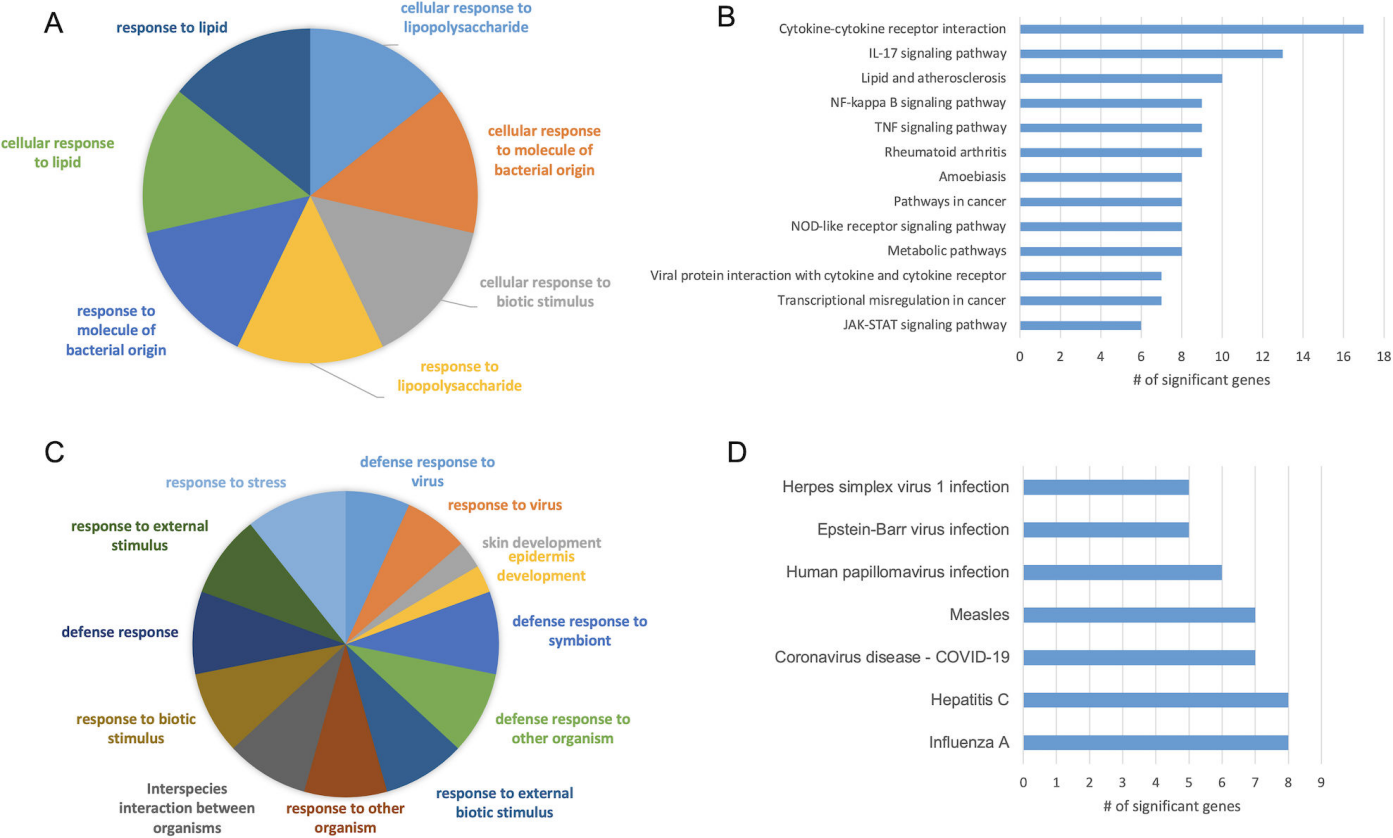
GO enrichment and KEGG pathways analysis of TIGK cells inoculated with *S. sanguinis* EMVs. (**A**) PANTHER GO-Slim Biological Process categories significantly enriched from genes upregulated by *S. sanguinis* EMV inoculation. (**B**) PANTHER GO-Slim Biological Process categories significantly enriched from genes downregulated by *S. sanguinis* EMV inoculation. (**C**) Top KEGG pathways enriched by genes upregulated by *S. sanguinis* EMV inoculation. (**D**) Top KEGG pathways enriched by genes downregulated by *S. sanguinis* EMV inoculation.

As *S. sanguinis* EMVs are internalized into TIGK gingival epithelial cells without causing cell disassociation and death, we aimed to determine global transcriptomic changes caused by EMV internalization. We sampled and sequenced the RNA from TIGK gingival epithelial cells 24 h post-inoculation with SK36 EMVs and compared the results to a PBS-inoculated control. Differential expression analysis showed that 140 genes were significantly changed at Benjamini-Hochberg adjusted *P* < 0.05, with 78 genes upregulated and 62 genes downregulated by EMV inoculation. The RNAseq statistics report is shown in Table S1, and a full list of significant genes and annotations can be found in Table S2, with a condensed list in Table 2. Gene Ontology (GO) enrichment analysis of genes upregulated by SK36 EMVs showed a clear immune signaling response, including categories like “response to biotic stimulus” and “cellular response to lipopolysaccharide” (Fig. 4A). Top Kyoto Encyclopedia of Genes and Genomes (KEGG) signaling pathways included “cytokine–cytokine receptor interaction,” “TNF signaling pathway,” and “JAK-STAT signaling pathway.” Included in these categories are significantly upregulated genes like IL-6 (+3.0-fold), IL-32 (+2.1-fold), IL-1β (+2.3-fold), and IL-24 (+2.7-fold). There are also several additional upregulated cytokines, including CXCL3 (+2.3-fold), CXCL8 (+2.-4fold), and colony stimulating factor 3 (3.4-fold).

**TABLE 2:** Condensed list of DEGs

Gene name	Full name	Log2FoldChange PBS/SK36MV	Padj	Gene name	PANTHER family/subfamily	PANTHER protein class
IL1R2	Interleukin-1 receptor type 2	−4.65	0.00	Interleukin-1 receptor type 2;IL1R2;PTN002484217;orthologs	INTERLEUKIN-1 RECEPTOR TYPE 2 (PTHR11890:SF3)	Transmembrane signal receptor(PC00197)
ACOD1	Aconitate decarboxylase 1	−3.55	0.00	Cis-aconitate decarboxylase;ACOD1;PTN002497709;orthologs	CIS-ACONITATE DECARBOXYLASE (PTHR16943:SF11)	Dehydratase(PC00091)
CSF3	Colony-stimulating factor 3	−3.50	0.00	Granulocyte colony-stimulating factor;CSF3;PTN002471210;orthologs	GRANULOCYTE COLONY-STIMULATING FACTOR (PTHR10511:SF2)	
NPTX1	Neuronal pentraxin-1	−3.04	0.02	Neuronal pentraxin-1;NPTX1;PTN002499349;orthologs	NEURONAL PENTRAXIN-1 (PTHR19277:SF24)	Scaffold/adaptor protein(PC00226)
IL6	Interleukin-6	−3.01	0.00	Interleukin-6;IL6;PTN002554292;orthologs	INTERLEUKIN-6 (PTHR48406:SF1)	
IL33	Interleukin-33	−2.88	0.00	Interleukin-33;IL33;PTN002501361;orthologs	INTERLEUKIN-33 (PTHR21114:SF0)	
IL24	Interleukin-24	−2.73	0.00	Interleukin-24;IL24;PTN008935621;orthologs	INTERLEUKIN-24 (PTHR48394:SF3)	
ALDH1A3	Aldehyde dehydrogenase 1 family member A3	−2.71	0.00	Aldehyde dehydrogenase family 1 member A3;ALDH1A3;PTN002482030;orthologs	ALDEHYDE DEHYDROGENASE FAMILY 1 MEMBER A3 (PTHR11699:SF209)	Dehydrogenase(PC00092)
CXCL1	C-X-C motif chemokine ligand 1	−2.55	0.00	Growth-regulated alpha protein;CXCL1;PTN002468774;orthologs	GROWTH-REGULATED ALPHA PROTEIN (PTHR12015:SF194)	Cytokine(PC00083)
BCL2A1	BCL-2 protein A1	−2.52	0.05	Bcl-2-related protein A1;BCL2A1;PTN002477649;orthologs	BCL-2-RELATED PROTEIN A1 (PTHR11256:SF10)	
MMP9	Matrix metallopeptidase-9	−2.49	0.02	Matrix metalloproteinase-9;MMP9;PTN002469000;orthologs	MATRIX METALLOPROTEINASE-9 (PTHR10201:SF30)	Metalloprotease(PC00153)
CXCL3	C-X-C motif chemokine ligand 3	−2.48	0.00	C-X-C motif chemokine 3;CXCL3;PTN002468775;orthologs	C-X-C MOTIF CHEMOKINE 3 (PTHR12015:SF210)	Cytokine(PC00083)
IL36G	Interleukin-36 gamma	−2.47	0.00	Interleukin-36 gamma;IL36G;PTN002467818;orthologs	INTERLEUKIN-36 GAMMA (PTHR10078:SF27)	Interleukin superfamily(PC00128)
CXCL8	C-X-C motif chemokine ligand 8	−2.45	0.00	Interleukin-8;CXCL8;PTN002468766;orthologs	INTERLEUKIN-8 (PTHR12015:SF211)	Cytokine(PC00083)
IL1B	Interleukin-1 beta	−2.38	0.00	Interleukin-1 beta;IL1B;PTN002552415;orthologs	INTERLEUKIN-1 BETA (PTHR10078:SF30)	Interleukin superfamily(PC00128)
MMP1	Matrix metallopeptidase 1	−2.34	0.00	Interstitial collagenase;MMP1;PTN002469008;orthologs	INTERSTITIAL COLLAGENASE (PTHR10201:SF151)	Metalloprotease(PC00153)
ANGPTL4	Angiopoietin-like 4	−2.19	0.00	Angiopoietin-related protein 4;ANGPTL4;PTN002499054;orthologs	ANGIOPOIETIN-RELATED PROTEIN 4 (PTHR19143:SF256)	Intercellular signal molecule(PC00207)
IL32	Interleukin-32	−2.11	0.00	Interleukin-32;IL32;PTN008935850;orthologs	INTERLEUKIN-32 (PTHR48402:SF1)	
IL23A	Interleukin-23 subunit alpha	−1.92	0.00	Interleukin-23 subunit alpha;IL23A;PTN002496783;orthologs	INTERLEUKIN-23 SUBUNIT ALPHA (PTHR15947:SF0)	Interleukin superfamily(PC00128)
TRAF1	TNF receptor-associated factor 1	−1.82	0.00	TNF receptor-associated factor 1;TRAF1;PTN002468275;orthologs	TNF RECEPTOR-ASSOCIATED FACTOR 1 (PTHR10131:SF96)	Scaffold/adaptor protein(PC00226)
SOCS3	Suppressor of cytokine signaling 3	−1.78	0.00	Suppressor of cytokine signaling 3;SOCS3;PTN002549627;orthologs	SUPPRESSOR OF CYTOKINE SIGNALING 3 (PTHR10155:SF11)	Kinase modulator(PC00140)
INHBA	Inhibin subunit beta A	−1.56	0.00	Inhibin beta A chain;INHBA;PTN002483773;orthologs	INHIBIN BETA A CHAIN (PTHR11848:SF133)	Growth factor(PC00112)
SOD2	Superoxide dismutase 2	−1.52	0.00	Superoxide dismutase [Mn], mitochondrial;SOD2;PTN002478665;orthologs	SUPEROXIDE DISMUTASE [MN], MITOCHONDRIAL (PTHR11404:SF41)	Oxidoreductase(PC00176)
CXCL2	C-X-C motif chemokine ligand 2	−1.50	0.00	C-X-C motif chemokine 2;CXCL2;PTN002468776;orthologs	C-X-C MOTIF CHEMOKINE 2 (PTHR12015:SF213)	Cytokine(PC00083)
HCAR2	Hydroxycarboxylic acid receptor 2	−1.48	0.01	Hydroxycarboxylic acid receptor 2;HCAR2;PTN002517336;orthologs	HYDROXYCARBOXYLIC ACID RECEPTOR 2 (PTHR46048:SF6)	G-protein coupled receptor(PC00021)
PHLDA1	Pleckstrin homology-like domain family A member 1	−1.41	0.00	Pleckstrin homology-like domain family A member 1;PHLDA1;PTN002496074;orthologs	PLECKSTRIN HOMOLOGY-LIKE DOMAIN FAMILY A MEMBER 1 (PTHR15478:SF4)	
TNFAIP3	TNF alpha-induced protein 3	−1.40	0.00	Tumor necrosis factor alpha-induced protein 3;TNFAIP3;PTN002491478;orthologs	TUMOR NECROSIS FACTOR ALPHA-INDUCED PROTEIN 3 (PTHR13367:SF3)	Cysteine protease(PC00081)
DAB2	DAB adaptor protein 2	−1.35	0.00	Disabled homolog 2;DAB2;PTN002477405;orthologs	DISABLED HOMOLOG 2 (PTHR47695:SF5)	
IL1A	Interleukin-1 alpha	−1.33	0.03	Interleukin-1 alpha;IL1A;PTN002556541;orthologs	INTERLEUKIN-1 ALPHA (PTHR10078:SF33)	Interleukin superfamily(PC00128)
NFKB2	Nuclear factor kappa B subunit 2	−1.24	0.05	Nuclear factor NF-kappa-B p100 subunit;NFKB2;PTN002516582;orthologs	NUCLEAR FACTOR NF-KAPPA-B P100 SUBUNIT (PTHR24169:SF21)	Rel homology transcription factor(PC00252)
S100A9	S100 calcium-binding protein A9	−1.10	0.05	Protein S100-A9;S100A9;PTN002481270;orthologs	PROTEIN S100-A9 (PTHR11639:SF79)	Calmodulin related(PC00061)
DTX3L	Deltex E3 ubiquitin ligase 3L	1.11	0.01	E3 ubiquitin-protein ligase DTX3L;DTX3L;PTN002489146;orthologs	E3 UBIQUITIN-PROTEIN LIGASE DTX3L (PTHR12622:SF41)	Ubiquitin-protein ligase(PC00234)
PARP14	Poly(ADP-ribose) polymerase family member 14	1.13	0.03	Protein mono-ADP-ribosyltransferase PARP14;PARP14;PTN002494687;orthologs	PROTEIN MONO-ADP-RIBOSYLTRANSFERASE PARP14 (PTHR14453:SF89)	
TRIM14	Tripartite motif containing 14	1.15	0.01	Tripartite motif-containing protein 14;TRIM14;PTN002553115;orthologs	TRIPARTITE MOTIF-CONTAINING PROTEIN 14 (PTHR24103:SF595)	Ubiquitin-protein ligase(PC00234)
EIF2AK2	Eukaryotic translation initiation factor 2 alpha kinase 2	1.15	0.00	Interferon-induced, double-stranded RNA-activated protein kinase;EIF2AK2;PTN002475930;orthologs	INTERFERON-INDUCED, DOUBLE-STRANDED RNA-ACTIVATED PROTEIN KINASE (PTHR11042:SF163)	Non-receptor serine/threonine protein kinase(PC00167)
IFI6	Interferon alpha-inducible protein 6	1.17	0.03	Interferon alpha-inducible protein 6;IFI6;PTN002497697;orthologs	INTERFERON ALPHA-INDUCIBLE PROTEIN 6 (PTHR16932:SF25)	
SAMD9	Sterile alpha motif domain containing 9	1.17	0.02	Sterile alpha motif domain-containing protein 9;SAMD9;PTN002497105;orthologs	STERILE ALPHA MOTIF DOMAIN-CONTAINING PROTEIN 9 (PTHR16155:SF17)	
IFIH1	Interferon-induced with helicase C domain 1	1.19	0.02	Interferon-induced helicase C domain-containing protein 1;IFIH1;PTN002493747;orthologs	INTERFERON-INDUCED HELICASE C DOMAIN-CONTAINING PROTEIN 1 (PTHR14074:SF14)	
PLSCR1	Phospholipid scramblase 1	1.26	0.04	Phospholipid scramblase 1;PLSCR1;PTN002509940;orthologs	PHOSPHOLIPID SCRAMBLASE 1 (PTHR23248:SF38)	Transporter(PC00227)
TRIM22	Tripartite motif containing 22	1.34	0.04	E3 ubiquitin-protein ligase TRIM22;TRIM22;PTN002515828;orthologs	E3 UBIQUITIN-PROTEIN LIGASE TRIM22 (PTHR24103:SF650)	Ubiquitin-protein ligase(PC00234)
OAS2	2'-5'-oligoadenylate synthetase 2	1.43	0.00	2'-5'-oligoadenylate synthase 2;OAS2;PTN002477651;orthologs	2'-5′-OLIGOADENYLATE SYNTHASE 2 (PTHR11258:SF3)	Nucleotidyltransferase(PC00174)
PARP9	Poly(ADP-ribose) polymerase family member 9	1.44	0.00	Protein mono-ADP-ribosyltransferase PARP9;PARP9;PTN002494684;orthologs	PROTEIN MONO-ADP-RIBOSYLTRANSFERASE PARP9 (PTHR14453:SF70)	
IFIT3	Interferon-induced protein with tetratricopeptide repeats 3	1.52	0.02	Interferon-induced protein with tetratricopeptide repeats 3;IFIT3;PTN002469627;orthologs	INTERFERON-INDUCED PROTEIN WITH TETRATRICOPEPTIDE REPEATS 3 (PTHR10271:SF3)	Defense/immunity protein(PC00090)
STAT1	Signal transducer and activator of transcription 1	1.55	0.00	Signal transducer and activator of transcription 1-alpha_beta;STAT1;PTN002483235;orthologs	SIGNAL TRANSDUCER AND ACTIVATOR OF TRANSCRIPTION 1-ALPHA_BETA (PTHR11801:SF18)	Rel homology transcription factor(PC00252)
XAF1	XIAP-associated factor 1	1.62	0.00	XIAP-associated factor 1;XAF1;PTN002497361;orthologs	XIAP-ASSOCIATED FACTOR 1 (PTHR16295:SF17)	
RSAD2	Radical S-adenosyl methionine domain containing 2	1.64	0.01	Radical S-adenosyl methionine domain-containing protein 2;RSAD2;PTN002501864;orthologs	RADICAL S-ADENOSYL METHIONINE DOMAIN-CONTAINING PROTEIN 2 (PTHR21339:SF0)	
OAS1	2'-5'-oligoadenylate synthetase 1	1.85	0.00	2'-5'-oligoadenylate synthase 1;OAS1;PTN002477654;orthologs	2'-5′-OLIGOADENYLATE SYNTHASE 1 (PTHR11258:SF13)	Nucleotidyltransferase(PC00174)
USP18	Ubiquitin-specific peptidase 18	1.92	0.00	Ubl carboxyl-terminal hydrolase 18;USP18;PTN002550882;orthologs	UBL CARBOXYL-TERMINAL HYDROLASE 18-RELATED (PTHR24006:SF796)	Cysteine protease(PC00081)
MX1	MX dynamin-like GTPase 1	2.06	0.00	Interferon-induced GTP-binding protein Mx1;MX1;PTN002480286;orthologs	INTERFERON-INDUCED GTP-BINDING PROTEIN MX1 (PTHR11566:SF217)	Membrane traffic protein(PC00150)
DSG1	Desmoglein-1	2.08	0.00	Desmoglein-1;DSG1;PTN002513167;orthologs	DESMOGLEIN-1 (PTHR24025:SF9)	Cadherin(PC00057)
DAPK1	Death-associated protein kinase 1	2.15	0.00	Death-associated protein kinase 1;DAPK1;PTN002550499;orthologs	DEATH-ASSOCIATED PROTEIN KINASE 1 (PTHR24342:SF17)	Non-receptor serine/threonine protein kinase(PC00167)
MX2	MX dynamin-like GTPase 2	2.23	0.00	Interferon-induced GTP-binding protein Mx2;MX2;PTN002480285;orthologs	INTERFERON-INDUCED GTP-BINDING PROTEIN MX2 (PTHR11566:SF46)	Membrane traffic protein(PC00150)
IFI44L	Interferon-induced protein 44-like	2.40	0.00	Interferon-induced protein 44-like;IFI44L;PTN002494424;orthologs	INTERFERON-INDUCED PROTEIN 44-LIKE (PTHR14241:SF2)	
CXCL14		2.47	0.02	C-X-C motif chemokine 14;CXCL14;PTN002495627;orthologs	C-X-C MOTIF CHEMOKINE 14 (PTHR12015:SF191)	Cytokine(PC00083)
IFIT1	Interferon-induced protein with tetratricopeptide repeats	2.50	0.00	Interferon-induced protein with tetratricopeptide repeats 1;IFIT1;PTN002469623;orthologs	INTERFERON-INDUCED PROTEIN WITH TETRATRICOPEPTIDE REPEATS 1 (PTHR10271:SF16)	Defense/immunity protein(PC00090)
ELN	Elastin	2.57	0.02	Elastin;ELN;PTN002513045;orthologs	ELASTIN (PTHR24018:SF5)	Structural protein(PC00211)
FLG	Filaggrin	2.61	0.00	Filaggrin;FLG;PTN002503125;orthologs	FILAGGRIN (PTHR22571:SF51)	Cytoskeletal protein(PC00085)
DSC1	Desmocollin 1	2.98	0.00	Desmocollin-1;DSC1;PTN002513170;orthologs	DESMOCOLLIN-1 (PTHR24025:SF8)	Cadherin(PC00057)

GO enrichment analysis of genes downregulated by SK36 EMVs also indicated an immune response, with significant categories including “Defense response to virus,” “defense response to other organism,” and “response to stress.” In accordance with this, several significantly downregulated genes have been implicated in interferon signaling, including IFIT1/2/3 (−2.5-, −1.5-, −1.5-fold) and MX1/2 (–2.1, −2.2-fold) among others. Other categories significantly enriched suggested alterations in cell development, including “skin development” and “epidermal cell differentiation.” There were several genes significantly downregulated in these categories known to be involved in keratinization (KRT1/4/75, with –1.5, −3.2, and –1.6-fold change). Additionally, desmosomal cadherins DSC1 and DSG1 were downregulated –3.0 and –2.1-fold, respectively. Other downregulated genes of interest that did not fit into a GO enrichment category included two apoptosis-related genes, DAPK1 (death-associated protein kinase 1), a positive regulator of apoptosis (−2.5-fold), and XAF1 (XIAP-associated factor 1), which inhibits XIAP, a negative regulator of apoptosis (−1.5-fold).

**Fig 5:**
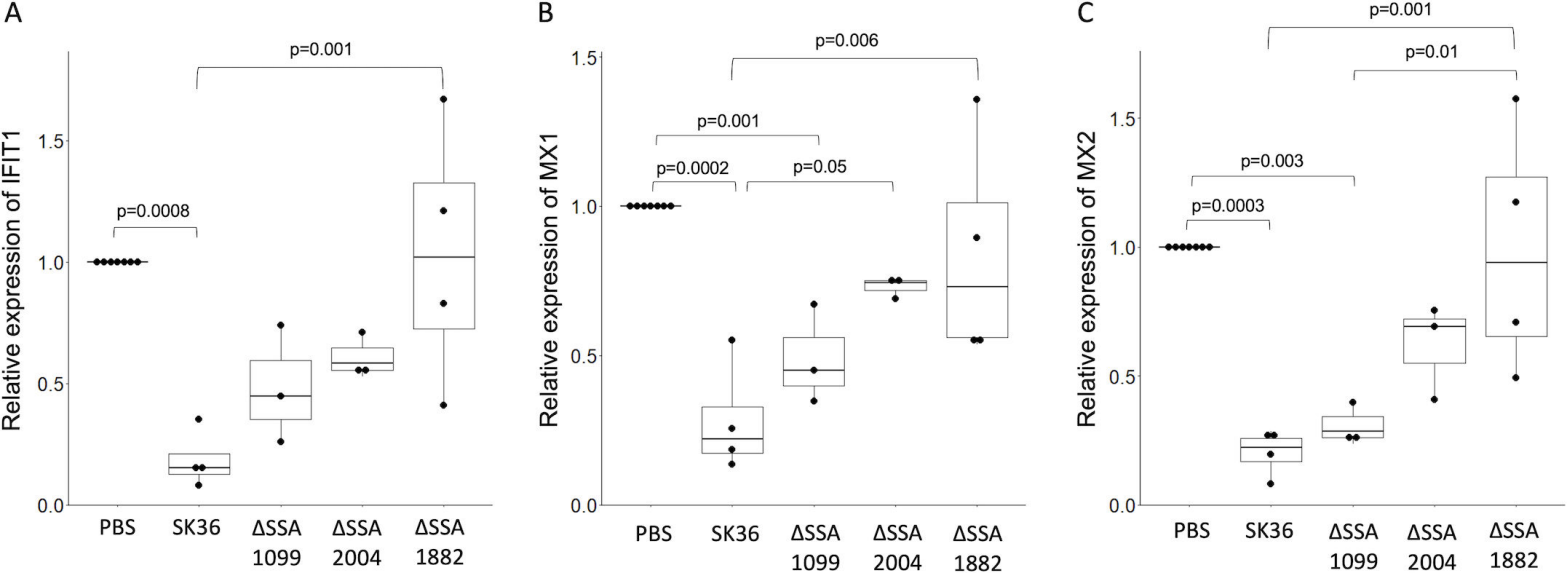
Inoculation of TIGK cells with either ΔSSA2004 or ΔSSA1882 EMVs shows no effect on the expression of interferon signaling genes. Boxplots show relative expression of (**A**) IFIT1, (**B**) MX1, and (**C**) MX2 24 h after inoculation with either PBS, EMVs from SK36, ΔSSA1099, ΔSSA2004, or ΔSSA1882 (10^11^ particles per sample). Each dot represents one biological replicate, with GAPDH used as an internal control. Significant differences are specified with brackets and labeled with *P* values.

Quantitative reverse transcriptase PCR was performed to independently validate these results. TIGK cells were inoculated with a 10-fold dilution series of SK36 EMVs (10^11^–10^7^ particles per sample). In agreement with the transcriptomic data, we detected a significant reduction in gene expression for IFIT1, MX1, MX2, DSC1, DSG1, and KRT1 in samples inoculated with 10^11^ and 10^10^ SK36 EMVs (Fig. 5A through C). These effects dissipated when <10^9^ EMVs were applied (Fig. S3). These results suggest that inoculation with SK36 EMVs will induce one type of immune response while inhibiting another in a dose-dependent manner.

To see if the expression patterns of interferon signaling genes were altered, when exposed to EMVs from the deletion mutants, dilution series of deletion mutants ΔSSA1099, ΔSSA1882, and Δ*zmpB* were inoculated alongside EMVs from wild-type SK36 onto TIGK cells with a range of 10^11^–10^9^ particles per sample. Higher dilutions of EMVs from SK36 and ΔSSA1099 triggered a significant downregulation of one or more interferon signaling genes, while EMVs from ΔSSA1882 and ΔSSA2004 did not show a significant effect compared to the PBS control (Fig. 5).

### *S. sanguinis* SK36 EMVs inhibit production of MMP2 and increase production of MMP9

**Fig 6:**
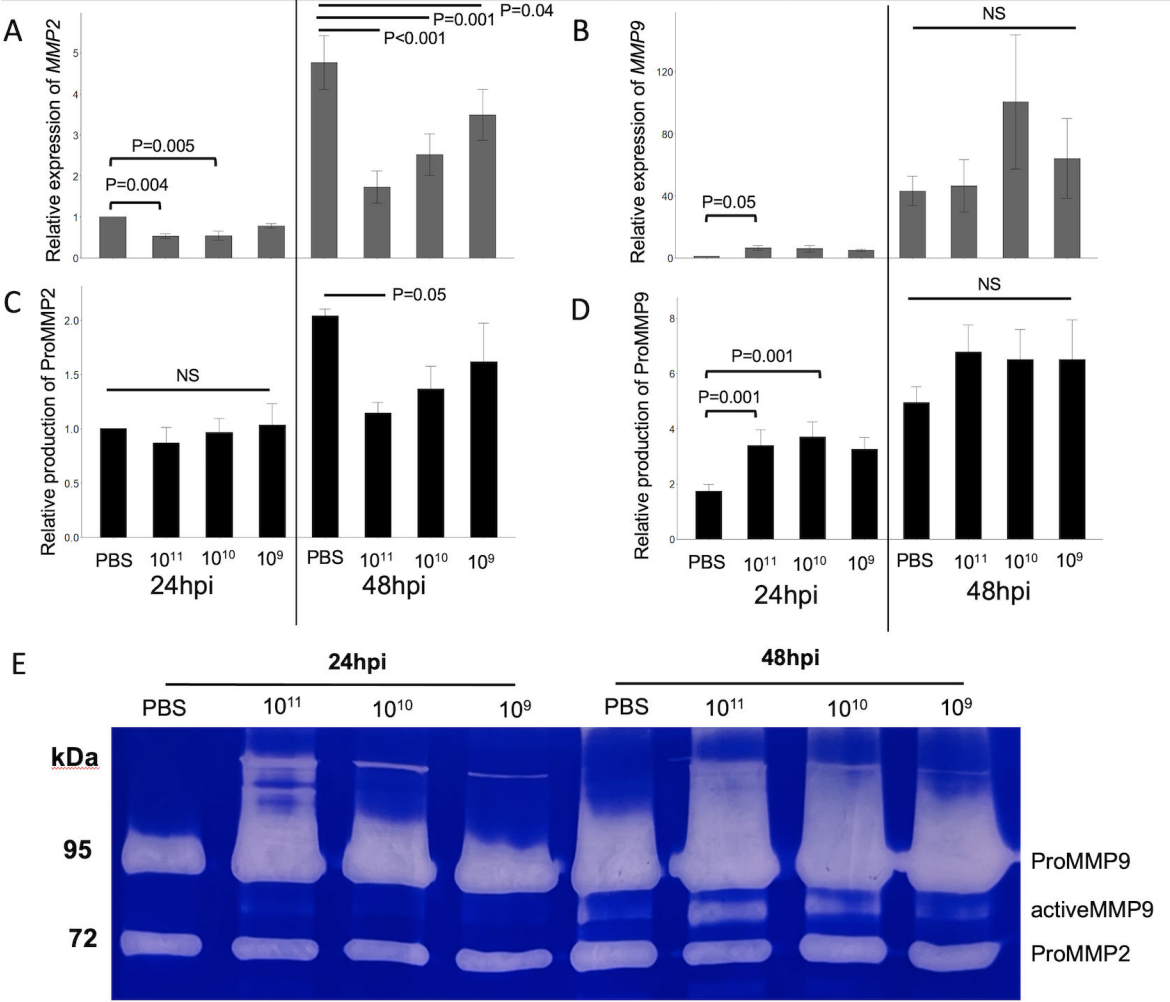
Gene expression and production of matrix metalloproteinases MMP2/9. (**A**) Expression of MMP2 24 and 48 hpi by SK36 EMVs. (**B**) Expression of MMP9 24 and 48 hpi after adding SK36 EMVs. Gelatin zymography of gingival epithelial cells, 24 and 48 hpi with SK36 EMVs. Gene expression is normalized relative to GAPDH. (**C**) Band size of latent (Pro)MMP2 (72 kDa), relative to ProMMP2 from GECs 24 hpi by PBS control. (**D**) Band size of latent (Pro)MMP9 (95 kDa), relative to ProMMP2 from GECs 24 hpi by PBS control. All band sizes were normalized by quantity of total protein extracted from individual samples. (**E**) Representative photo of gelatin zymography, quantity of inoculum, and time post-inoculation is labeled. All bars represent the averages of three biological replicates, and error bars indicate standard error. *P* values of significant differences are labeled. NS, not significant.

Gene expression analysis on TIGK cells showed a significant (−0.5-fold) downregulation of MMP2 within 24 h of inoculation by 10^11^ and 10^10^ particles of SK36 EMVs. The expression of MMP2 continued to decrease by 48 hpi, in which all concentrations tested (10^11^ down to 10^9^ particles per sample) were significantly decreased compared to the PBS control (Fig. 6A). In contrast, TIGK cells showed a significant upregulation of MMP9 24 hpi by *S. sanguinis* EMVs (10^11^ particles), but this effect dissipated by 48 hpi (Fig. 6B). Gelatin zymography was performed to independently confirm the production of latent and active forms of MMP2/9 24 and 48 hpi (Fig. 6C). ImageJ analysis of band sizes shows a differing pattern of MMP2 and 9 from inoculated TIGK cells. At 24 hpi, a significant increase in latent MMP9 (ProMMP9) is seen in TIGK cells after inoculation by higher concentrations of SK36 EMVs (10^11^–10^10^ particles), while there was no significant difference in the production of ProMMP2 (Fig. 6D and E). The opposite pattern is seen at 48 hpi, as there is a significant decrease in the levels of ProMMP2 in TIGK cells inoculated with higher concentrations of SK36 EMVs, whereas there is no significant difference in the production of MMP9 between EMV- and PBS-inoculated cells (Fig. 6D and E). These results demonstrate that gelatinases MMP2 and 9 are differentially regulated by *S. sanguinis* EMVs in TIGK gingival epithelial cells.

### Pre-inoculation of TIGK cells with SK36 EMVs protects against cell disassociation and death after *P. gingivalis* OMV inoculation

**Fig 7:**
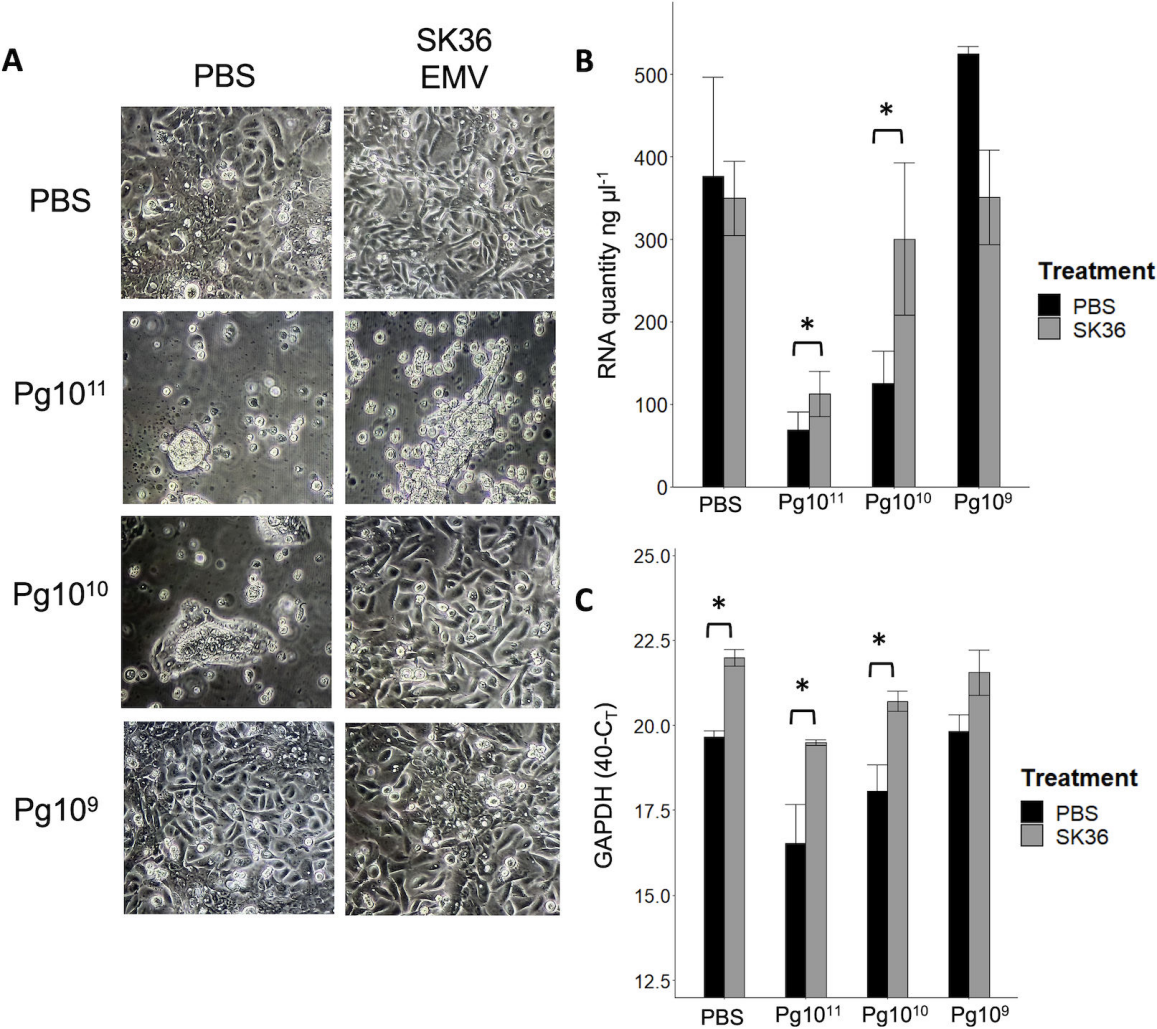
Pre-treatment with SK36 EMVs (10^11^ particles per sample) increases TIGK cell viability after *P. gingivalis* EMV inoculation. (**A**) Representative photos of TIGK cells pre-treated with either *S. sanguinis* EMVs (top row) or PBS control (bottom row), and then inoculated with PBS or a designated concentration of *P. gingivalis* 33277 OMVs. Photos taken 48 h after pre-treatment, 24 h after *P. gingivalis* OMV inoculation, 40× magnification. (**B**) Quantity of total RNA isolated from TIGK cells pre-treated with either *S. sanguinis* EMVs (gray bars) or PBS control (black bars). *P. gingivalis* OMV concentration is designated on the *x*-axis. (**C**) Inverse Ct value (40-Ct) of housekeeping gene GAPDH measured in RNA from TIGK cells pre-treated with either *S. sanguinis* EMVs (gray bars) or PBS control (black bars). *P. gingivalis* OMV concentration is designated on the *x*-axis. Bars represent the averages of three biological replicates, and error bars indicate standard error; asterisks designate significant differences (*P* < 0.05) between PBS- and SK36 EMV-treated cells for the specified *P. gingivalis* EMV inoculum levels.

**Fig 8:**
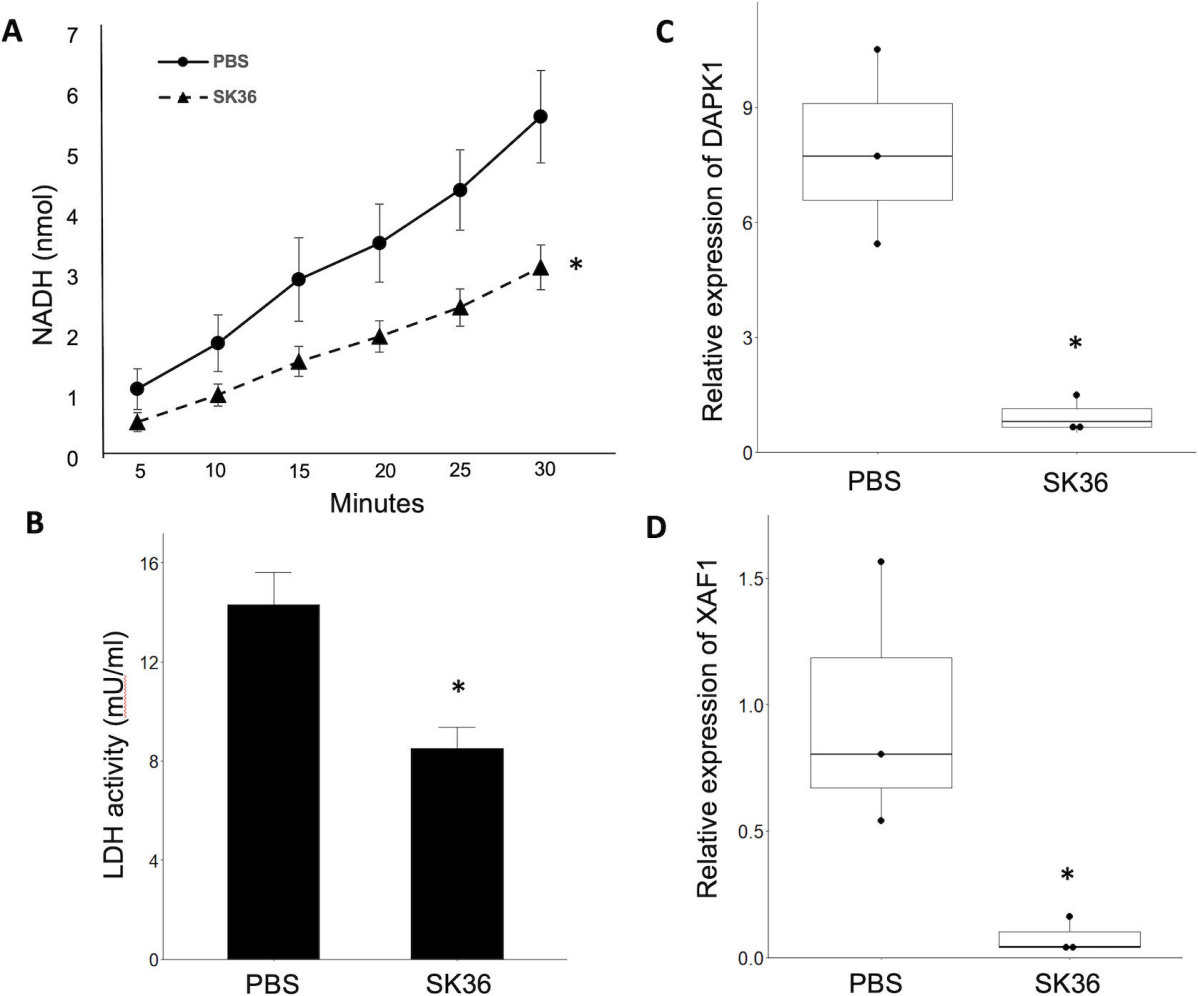
Assessment of cell death due to *P. gingivalis* OMVs after treatment with *S. sanguinis* EMVs. (**A**) Generation of NADH (nmol) by lactate dehydrogenase (LDH) in TIGK cell supernatant 24 h post-inoculation by *P. gingivalis* OMV (10^11^ OMV per sample), by cells pre-treated with either 10^11^ SK36 EMVs or a PBS control. (**B**) Calculation of LDH activity from PBS- or SK36 EMV-treated TIGK cells post-inoculation by *P. gingivalis* OMV (10^11^ particles per sample). Asterisk denotes a significant difference in the activity of LDH between SK36- and PBS-treated cells (*P* = 0.013). (**C**) Expression of death-associated protein kinase 1 (DAPK1) or (**D**) XIAP-associated factor 1 (XAF1) in cells inoculated with 10^11^ *P. gingivalis* OMVs 24 h after pre-treatment with either 10^11^ SK36 EMVs or a PBS control. Asterisks denote significance of *P* < 0.05 (DAPK1, *P* = 0.037; XAF1, *P* = 0.049).

Previous studies have demonstrated that the use of probiotic lactobacilli decreases the infective ability of *P. gingivalis* (18, 19). This, coupled with our results showing a decrease in the expression of apoptotic genes as well as decreased latent MMP2 in TIGK cells after *S. sanguinis* EMV inoculation, suggests that pre-treatment with *S. sanguinis* EMVs may prevent gingival epithelial cells from disassociation and death due to *P. gingivalis* OMV, which typically trigger apoptotic cell death (20). SK36 EMVs were inoculated onto TIGK cells, incubated for 24 h for internalization, and then inoculated with different concentrations of *P. gingivalis* 33277 OMVs (10^11^–10^8^ particles per sample). Representative images are shown in Figure 7A. We observed an increased occurrence of cell rounding and disassociation in *P. gingivalis* OMV-inoculated cells that were not pre-treated with SK36 EMVs. The viability of adhered cells was determined by measuring the total RNA isolated from samples (Fig. 7B), as well as the expression of the housekeeping gene GAPDH (Fig. 7C). Using both methods to quantify cell viability, we observed a significantly higher number of viable cells after inoculation with 10^11^ and 10^10^ *P. gingivalis* EMVs in TIGK cells pre-treated with *S. sanguinis* EMVs compared to PBS. To quantify cell death, we measured the activity of lactate dehydrogenase (LDH) in culture supernatants of cells inoculated by high concentrations of *P. gingivalis* OMVs (10^11^ per sample). LDH leaks from dead/damaged cells, which make the quantification of LDH activity a good representative of cell death. Quantification of NADH (a product of LDH activity) in *P. gingivalis* OMV-inoculated cells showed a lower generation over time in cells pre-treated with SK36 EMVs than a PBS-inoculated control (Fig. 8A). Calculations of the enzymatic activity from generation of NADH showed significant differences between PBS control-treated and SK36 EMV-treated cells (14.3 mU/mL vs 8.5 mU/mL, *P* = 0.013) (Fig. 8B). The expression of genes involved in apoptosis, DAPK1 and XAF1, was measured using qPCR (Fig. 8C and D). Cells that were pre-treated with SK36 EMVs prior to *P. gingivalis* OMV inoculation showed a significant decrease in the expression of both DAPK1 (*P* = 0.037) and XAF1 (*P* = 0.049) 24 hpi by *P. gingivalis* OMVs (10^11^ particles per sample). These results suggest that *S. sanguinis* EMVs confer a protective effect on gingival epithelial cells against *P. gingivalis* OMV, potentially by reducing apoptosis.

## DISCUSSION

The purpose of this work was to further characterize the immunostimulatory properties of extracellular membrane vesicles produced by the oral commensal *S. sanguinis*. We previously showed that *S. sanguinis* EMVs were internalized into gingival epithelial cells within 24 h of inoculation, and they elicited a rapid immune response that was comparable to both *S. sanguinis* whole cells as well as EMVs and whole cells of the pathobionts *F. alocis* and *P. gingivalis* (9). Here, we further characterized the changes that occur in gingival epithelial cells once *S. sanguinis* EMVs are internalized, and we also investigated the roles of specific proteins within *S. sanguinis* EMVs. We did replicate some of our studies using *S. sanguinis* whole cell inoculations (MOI 100) and found that expression patterns of several genes of interest (IFIT, MX1 and MX2, cadherins DSC1 and DSG1) followed the same expression patterns as cells inoculated with EMVs (Fig. S4).

Similarly, to what we observed in reference (9), SK36 EMVs were internalized into TIGK cells by 24 h post-inoculation. However, we observed significant differences in the degree of internalization of EMVs from two deletion mutants, ΔSSA1099 and ΔSSA1882. The internalization of EMVs derived from ΔSSA1099 was significantly lower compared to SK36 EMVs, as evidenced by the limited presence of fluorescent staining in only a few cells. Notably, the few cells that did exhibit staining were strongly fluorescent, possibly indicating internalization into damaged cells. This reduced internalization may be explained by the role of the SSA1099 protein as a pore-forming cytotoxin. The cytotoxic virulence factor VacA from *Helicobacter pylori* facilitates OMV entry into gastric epithelial cells through clathrin-mediated endocytosis, with VacA-deficient mutants having a much lower rate of entry (21). In contrast to ΔSSA1099, EMVs from ΔSSA1882 were internalized at much higher numbers than EMVs from SK36, with the potential explanation further discussed below.

The use of an ELISPOT-based cytokine assay revealed that EMVs from WT SK36 as well as deletion mutant strains ΔSSA1099 and Δ*zmp*B all elicit cytokine secretion from gingival epithelial cells 24 h post-inoculation. The quantity and type of cytokines produced did not vary among the EMVs collected from the different strains. This suggests that cytokine production may be a part of the rapid, innate immune signaling response by eukaryotic cells to a microbial conserved pattern present on the EMVs, or that the presence/absence of more potentially specialized virulence factors does not affect cytokine secretion. Along these lines, EMVs from unrelated species also trigger cytokine production, as demonstrated in the gram-positive pathobiont *Filifactor alocis* (22).

RNA sequencing of gingival epithelial cells inoculated with *S. sanguinis* EMVs had some of the common immunological hallmarks including upregulation of several cytokines and chemokines, which we would expect from nanoparticles of prokaryotic origin. We were particularly interested in genes that were downregulated in response to *S. sanguinis* EMVs. Among these, we identified genes that were potentially linked to anti-apoptotic pathways including XIAP-associated factor 1 (XAF1), which inhibits XIAP (X-linked inhibitor of apoptosis protein). As XIAP inhibits apoptosis by inhibiting caspase3 (23), we can hypothesize that downregulation of XAF1 has an overall negative effect on apoptosis. Another downregulated gene is Death-Associated Protein Kinase 1 (DAPK1), which is induced by interferon-gamma (IFN-gamma) and has been demonstrated to positively regulate both apoptotic and autophagic cell death (24). Also, among the significantly downregulated are genes involved in keratinization. These include Desmocollin 1 (DSC1) and Desmoglein (DSG1). DSC1 and DSG1 are desmosomal cadherins, which control cell–cell adhesion. As epidermal cells differentiate into keratinocytes, the expression of DSC1, DSG1, Keratin 1 (KRT1), and Fillagrin (FLG) all increase (25). According to our RNA sequencing and follow-up gene expression results, DSC1, DSG1, and KRT1 are all downregulated by SK36 EMVs in a dose-dependent manner, which suggests delayed differentiation of epidermal cells into terminal keratinocytes. A notable finding in our RNA sequencing results is the downregulation of several interferon signaling pathway genes. Interferon signaling is commonly seen in response to viral infection and has been shown to be upregulated after inoculation by bacterial cells and EMVs, both pathogenic (26) as well as commensals (27, 28). We selected three genes (IFIT1, MX1, and MX2) to verify their expression after inoculation by a dilution series of SK36 EMVs and found that inhibition of interferon signaling by SK36 EMVs occurs in a dose-dependent manner, with higher concentrations of SK36 EMVs showing a larger degree of inhibition. Inhibition of interferon signaling is commonly seen with viral infections (29); however, it is rarely reported with bacterial infections. To further study these results, we wanted to see if EMVs from our deletion mutants also triggered similar patterns in TIGK cells. Inoculation and subsequent expression of IFIT1, MX1, and MX2 exhibited a similar decrease in the expression with EMVs from ΔSSA1099 and Δ*zmpB* with SK36. However, all tested concentrations of EMVs from ΔSSA1882 yielded responses similar to the control PBS-inoculated TIGK cells. A question that remains is, how are *S. sanguinis* EMVs inhibiting interferon signaling? Based on the results from the ΔSSA1882 mutant, we hypothesize that the SSA1882 protein could be demonstrating protease activity against one or more interferons. *S. pyogenes* ScpA, an ortholog of SSA1882, was shown to cleave IFN-*gamma*, which inhibits downstream interferon signaling (30). If SSA1882 is cleaving one or more interferons, this would likely result in the inhibition of downstream signaling and reduced expression of IFIT1, MX1, and MX2. Conversely, the lack of SSA1882 in the deletion mutant would leave the pathway unchanged.

The lack of changes in interferon signaling after inoculation by ΔSSA1882 EMVs may also explain their increased internalization into gingival epithelial cells. Current models suggest that interferon signaling is initiated at the plasma membrane by the binding of an interferon to a receptor, followed by clathrin-dependent endocytosis (31). As clathrin-mediated endocytosis has been shown to be one of the main entries of EMVs (32), it could be that gingival epithelial cells with active interferon signaling are more efficiently internalizing ΔSSA1882 EMVs than those inoculated with SK36 WT EMVs. More experiments are needed to confirm both the entry mechanism of *S. sanguinis* EMVs as well as the protease activity of SSA1882.

A question that still remains is: how unique is the inhibition of interferon signaling among oral bacteria? If SSA1882 is a major regulatory effector in the inhibition of interferon signaling, we can predict that the oral commensal *Streptococcus gordonii*, which has three predicted S8 family serine peptidases, might also exhibit similar activity. Genome searches of another well-studied oral *Streptococcus*, the caries-associated *Streptococcus mutans*, revealed no similar predicted proteins. Further studies are needed to perform a detailed characterization of SSA1882’s activity and to determine whether these results are unique to commensal streptococci within the oral cavity.

A hallmark of early periodontal disease is the release of lytic enzymes by a range of cell types, including epithelial cells (33). These enzymes include matrix metalloproteinases (MMPs), which, in an activated form, degrade collagen and gelatin, leading to a loss of gingival attachment (34). We used quantitative reverse transcriptase PCR and gelatin zymography to quantify the expression of gelatinases MMP2/9 in gingival epithelial cells 24 and 48 hpi with SK36 EMVs. Our findings showed an increase in the expression and quantity of ProMMP9 at 24 hpi. In contrast, the expression of MMP2 decreased in cells inoculated with SK36 EMVs as early as 24 hpi and sustained at 48 hpi. Consistent with this observation, production of ProMMP2 was significantly decreased at 48 hpi, without observable active forms of MMP2. Previous studies have indicated that oral pathobionts can play a role in increasing MMP2 activity. While *P. gingivalis* exerts a negligible effect upon MMP2 expression (35), gingipains from *P. gingivalis* present in gingival crevicular fluid (GCF) are responsible for cleavage of ProMMP2 to its active form (36). Inoculation of periodontal fibroblasts with the pathobiont *T. denticola* increased levels of active MMP2, along with actin depolymerization and cell disassociation (2). Inoculation by *S. sanguinis* EMVs does not result in any visible forms of active MMP2, but we found that it did decrease the amounts of ProMMP2. Therefore, we can hypothesize that the presence of *S. sanguinis* EMVs results in a lower “pool” of ProMMP2 that could be converted to active MMP2 by a pathobiont, thereby reducing cell disassociation and tissue loss.

Based on the results from our transcriptomic and gelatin zymography work, we hypothesized that exposure to SK36 EMVs could enhance immune signaling in gingival epithelial cells and protect against *P. gingivalis* OMVs. Our previous study showed that inoculation with *P. gingivalis* OMVs caused cell detachment and death in TIGK cells (9). Cell detachment is a common approach to visualize and quantify stress in cultures of adherent cells (2). We used two RNA-based methods to quantify adherent cells: first, by measuring the total RNA from collected cells, and second, by measuring the abundance of GAPDH transcripts in each sample of total RNA. Measurement of the expression of ribosomal RNA has been used to quantify prokaryotic cells (37), and GAPDH is widely used as a standard for quantifying both nucleic acid and protein samples. Both total RNA and measurement of GAPDH show that TIGK cells pre-treated with SK36 EMVs showed reduced cell detachment and decreased LDH activity compared to control PBS-treated TIGK cells after inoculation by *P. gingivalis* OMVs, with significant differences between SK36 EMV pre-treated and a PBS control at each concentration of *P. gingivalis* OMVs. We do not yet know how SK36 EMVs confer production; it could be a combination of factors between a downregulation of pathways and processes that lead to cell detachment. Based on the decreased expression of apoptosis-related genes DAPK1 and XAF1 in cells pre-treated with SK36 EMVs, we hypothesize that pathways leading to apoptosis are reduced by SK36 EMVs. However, there could be other processes in play, such as the decreased production of proMMP2 and the downregulation of desmosomal cadherins, for example. In another system, cell detachment after inoculation by *T. denticola* is mediated by actin remodeling following the upregulation of GTPase RASA4 and an increase in MMP2 production (2). Another possibility is that excess or uninternalized SK36 EMVs in the supernatant may be inhibiting the *P. gingivalis* OMVs; however, an experiment in which we refreshed the cell media prior to *P. gingivalis* OMV inoculation (therefore removing uninternalized SK36 EMVs) yielded no difference in the outcome (data not shown). These results suggest that *S. sanguinis* EMVs may be exploited for therapeutic function. One important consideration is the timing of inoculation, which was not assessed in this study. Our experimental conditions were suboptimal for addressing these questions due to limited timepoints and the presence of dead or dying cells, which likely affected the results. Excessive “runaway” inflammation caused by constrained culture conditions may have exacerbated negative effects. A logical next step would be to use a murine model to test inflammation in gingival tissue through temporal inoculations of *P. gingivalis* and *S. sanguinis* EMVs. EMVs still remain a relatively unknown factor in microbial-host systems, as well as in more complex polymicrobial systems. After entering host cells, EMVs/OMVs have been shown to affect host cells at the epigenetic level (20). Epigenetic changes can have long-lasting effects on host cells and tissue, potentially lending either to protective effects, or severe inflammation and loss of tissue. Future studies will focus on the mechanisms by which SK36 EMVs confer protection to gingival epithelial cells, and whether differences in internalization seen with the deletion mutants affect the degree of protection against *P. gingivalis* OMVs.

## MATERIALS AND METHODS

### Bacterial strains, cells, and environmental conditions

Reference strain *Streptococcus sanguinis* SK36 and all mutants were cultured in brain-heart infusion (BHI) broth at 37°C, 5% CO_2_. *Porphyromonas gingivalis* ATCC 33277 was maintained on BHI agar augmented with 2% yeast extract, 5 µM hemin, and 0.5 µM menidione for under anaerobic (90% N_2_, 5% CO_2_, 5% H_2_) conditions. Immortalized normal human gingival keratinocyte cell line hTERT TIGKs (ATCC CRL3397) (38) were maintained in keratinocyte growth medium supplemented with epidermal growth factor, epinephrine, hydrocortisone, transferrin, insulin, and bovine pituitary extract (Lonza, KGM-Gold Bullet kit).

### Generation of bacterial deletion mutants

*S. sanguinis* deletion mutants Δ*aroB*, Δ*prtS*, and Δ*zmpB* were created using allelic replacement similar to reference (8), while ΔSSA1099 as created in reference (13) was generously donated by Dr. Bradley Jones at the University of Iowa. Fragments upstream and downstream of the specific gene were amplified using GoTaq Polymerase (Promega). Overlapping PCR (Accuprime Pfx DNA Polymerase, Invitrogen) was used to combine the PCR fragments flanking the kanamycin resistance gene aph3 (amplified from plasmid pJHI). *S. sanguinis* SK36 was cultured overnight in BHI media, then inoculated at 1:40 dilution into sterile BHI and grown to an OD600 = 0.05 to 0.08. The culture (1 mL) was mixed with 2 µL of competence stimulating peptide (1 mg mL-^1^) and the entire PCR reaction (~20 µL), then incubated for 2 h in a 37°C water bath. Successful transformants were selected on BHI agar containing kanamycin sulfate (300 µg mL^−1^), then verified using sequencing (Eurofins) and checking expression levels of specified genes using quantitative RT-PCR.

### Preparation and quantification of membrane vesicles

Isolation of EMVs followed the procedure described in reference (9). Overnight BHI cultures of *S. sanguinis* were adjusted to an OD_600_ = 1.5, and 1:40 vol was added into 250–1,000 mL Chemically Defined Medium (CDM) supplemented with 3.4% glucose and incubated for 18 h in aerobic conditions before EMV isolation. *P. gingivalis* was cultured in augmented BHI broth for 48 h (to OD_600_ > 0.7) before addition into 500 mL of augmented BHI broth, then incubated for an additional 48 h under anaerobic conditions. EMVs were isolated using a differential centrifugation procedure, starting with the removal of whole cells by centrifugation for 10 min at 4,000 rpm, followed by filtration of supernatants through a 45 µm cellulose pore (Fisher). Supernatants were concentrated ~20× using VivaSpin columns with 100 kDa cutoff (Sartorius), followed by ultracentrifugation at 35,000 rpm for 2 h, and the resulting pellet was washed by resuspension in 1× PBS and ultracentrifuged a second time under the same condition. Pellets were resuspended in 1 mL of 1× PBS and stored at either 4°C for use within 72 h or −80°C for long-term storage.

Vesicles were quantified with NTA using ZetaView (Particle Metrix, Germany). For each measurement, 11 cell positions were scanned with 60 frames per position captured. The resulting videos were analyzed by the in-build ZetaView Software version 8.05.12 with specific analysis parameters: laser wavelength, 488 nm; filter wavelength, scatter ; maximum particle size, 1,000; minimum particle size, 10; minimum particle brightness, 20.

### Staining and imaging of EMV internalization into gingival epithelial cells

The *S. sanguinis* EMV internalization assay was performed similarly to reference (39), following the same protocol as written in reference (9). Briefly, *S. sanguinis* wild-type and mutant EMVs were isolated from 500 mL of CDM + 3.4% glucose, labeled as per the manufacturer’s protocol with Vybrant-DiO (Invitrogen), washed 3× with 1× PBS through ultracentrifugation (1 h at 35,000 rpm, 4°C), and incubated overnight at 4°C to fully resuspend the pellet. TIGK CRL3397 cells were seeded onto 6-well plates containing coverslips at a concentration of 5 × 10^5^ cells per well. Cells were inoculated with 10^11^ labeled vesicles, or a similar volume of 1× PBS and incubated for 24 h. Cells were treated with stripping buffer (500 µM NaCl and 0.5% acetic acid in deionized water [pH 3]) for 45 s to remove plasma membrane-bound EMVs. Cells were then fixed with 4% paraformaldehyde (PFA; Electron Microscopy Services) for 20 min, followed by quenching with an equal volume of 1% (wt/wt) bovine serum albumin (BSA) in 1× PBS for 5 min. Cells were permeabilized for 1 min with 0.1% Triton X-100 in 1× PBS. To visualize F-actin, cells were stained with 10:200 Texas Red-X phallodin in 1× PBS (Thermo Fisher Scientific, Waltham, MA, USA) according to the manufacturer’s instructions. Cells were washed two to three times with 1× PBS following each step. Coverslips were air dried in a laminar flow hood for 5 min, then placed cell-side down onto a slide containing a drop of ProLong Gold Antifade reagent with 4’,6-diamidino-2-phenylindole (DAPI) (Invitrogen) and incubated in the dark for 18 h. Fixed, stained cells were visualized on a Zeiss Laser-Scanning Confocal Microscope with Airyscan.2, and images were processed using Fiji (ImageJ) (40).

### Measurement of cytokines and chemokines from gingival epithelial cells

TIGK CRL3977 cells were inoculated with 10^11^ EMVs similar to those described above and incubated for 24 h in aerobic conditions. Cell supernatant was centrifuged for 10 min at 4,000 rpm to pellet any cells, and cytokines and chemokines were detected from 1 mL of clarified supernatant using a Human Cytokine Array (Bio-Techne R&D Systems) according to the manufacturer’s protocol. Arrays were visualized using chemiluminescent detection, and dot intensities were quantified using ImageJ.

### RNA extraction and quantitative reverse transcriptase PCR

Cell pellets were resuspended in 1 mL of TRIzol reagent and cycled through two bead-beating sessions of 1 min at 8,300 rpm, followed by chloroform extraction and isopropanol precipitation. Samples were incubated with DNAseI for 2 h at 37°C to degrade genomic DNA, followed by a column clean-up using the RNeasy Mini Kit (Qiagen). cDNA synthesis was carried out with the qScript cDNA synthesis kit (Quanta Biosciences) according to the manufacturer’s specifications. Quantitative RT-PCR was carried out using a protocol as described in reference (4).

### Gelatin zymography

Gelatin zymography was carried out similarly to the Abcam protocol (Abcam.com) and as cited in reference (2). One milliliter of clarified supernatant per sample was concentrated 10× using Biomax centrifugal filters with a 10 kDa cutoff (Sigma). Ten microliters of concentrated sample was combined with 5× non-reducing sample buffer (250 mM Tris, 0.8% SDS, 40% glycerol, 0.05% Bromophenol blue) and run on a 1 mm thick 10% SDS-PAGE with 0.1% gelatin. After sample separation, gels were washed for 2 × 30 min in renaturing buffer (2.5% Triton X-100, 50 mM Tris-HCl, 5 mM CaCl_2_, 1 µM ZnCl_2_) to remove SDS, then incubated for 18 h at 37°C in reaction buffer (1% Triton X-100, 50 mM Tris-HCl, 5 mM CaCl_2_, 1 µM ZnCl_2_). Gels were stained in 0.05% Bromophenol blue for approximately 1 h, then cleared in 10% acetic acid and 40% methanol until cleared bands could be visualized. Quantification of band size was carried out using ImageJ software.

### RNA sequencing and analysis

Total RNA was extracted as detailed above, treated with DNaseI, and sent for library preparation, sequencing, and analysis at Zymo Research. rRNA was depleted, and libraries were prepared using Zymo-Seq RiboFree total RNA kit. Sequencing was performed on a NovaSeq 6000 (Illumina), followed by demultiplexing and quality checking (QC) using FastQC. Sequences were processed using Samtools and aligned to Homo sapiens genome (GRCh38) using STAR (41). Sequence normalization and differential expression analysis was performed using Deseq2 (42).

## Data Availability

RNA sequencing raw data and analysis are deposited at the Gene Expression Omnibus (GEO) database under GSE288367.

## SUPPLEMENTAL MATERIAL

Supplemental figures iai.00535-24-s0001.pdf Fig. S1 to S4.

Supplemental material iai.00535-24-s0002.docx Supplemental table legends.

Table S1 iai.00535-24-s0003.xlsx General statistics of RNAseq data set from TIGK cells inoculated with SK36 EMVs, 24 hpi.

Table S2 iai.00535-24-s0004.xlsx List of all significantly differentially regulated genes and DEseq2 output (Padj < 0.05) from RNAseq data set from TIGK cells inoculated with SK36 EMVs, 24 hpi.

Table S3 iai.00535-24-s0005.xlsx List and sequences of all primers used.

ASM does not own the copyrights to Supplemental Material that may be linked to, or accessed through, an article. The authors have granted ASM a non-exclusive, world-wide license to publish the Supplemental Material files. Please contact the corresponding author directly for reuse.
