# Supplementary material for: Immunostimulatory effects of Streptococcus sanguinis extracellular membrane vesicles protect oral gingival epithelial cells from periodontal pathobiont damage
Source: Infect Immun. 2025 Feb 19;93(3):e00535-24. doi: 10.1128/iai.00535-24 (PMC11895462; doi:10.1128/iai.00535-24)
Supplement: Supplemental figures — Fig. S1 to S4. [file iai.00535-24-s0001.pdf]

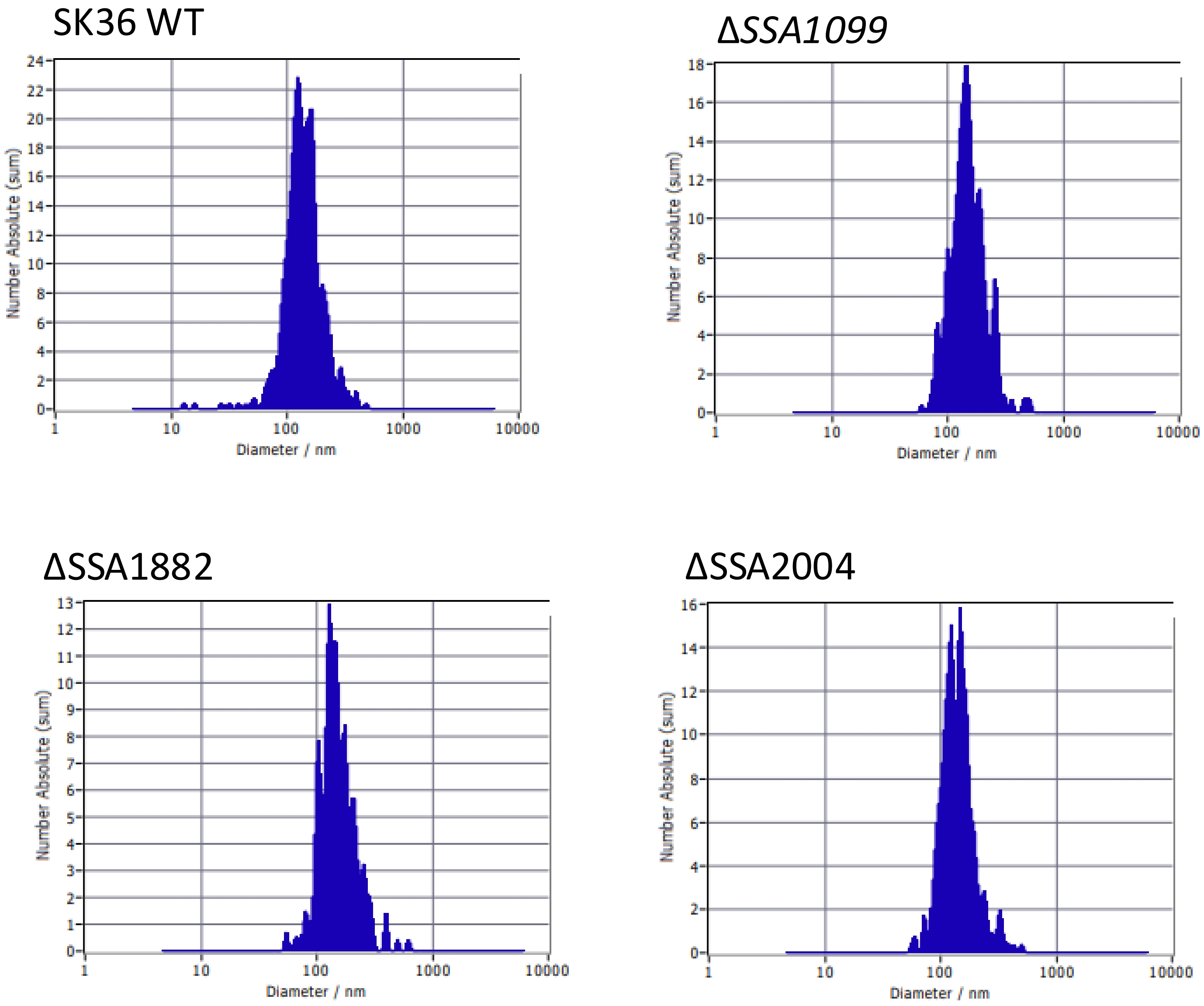

Fig S1: Size distribution analysis comparison of EMVs from SK36 WT and deletion mutants.

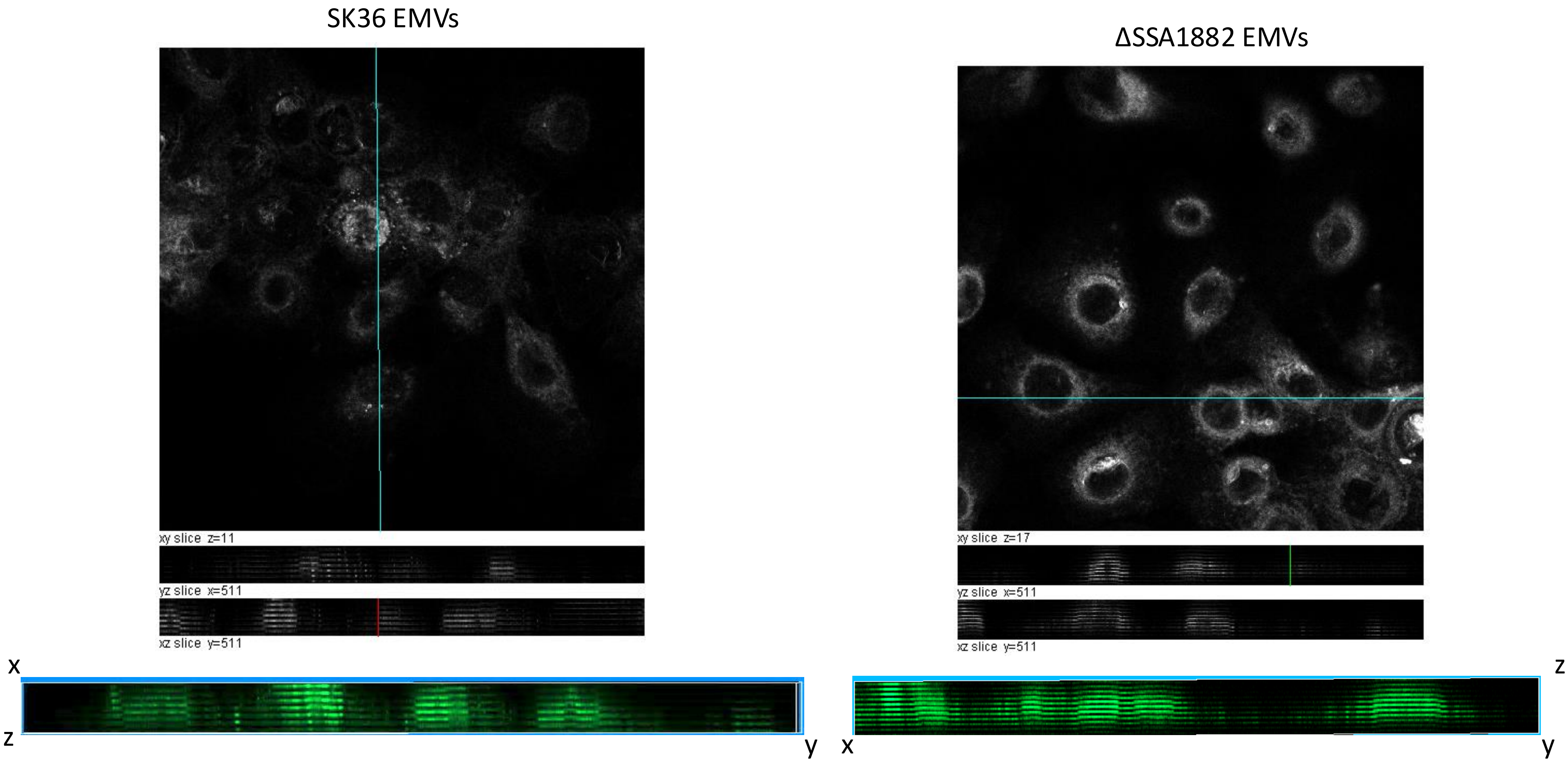

Fig. S2: Representative images of z-stacks showing internalization of SK36 and ΔSSA1882 EMVs into gingival epithelial cells (TIGK CRL3397) at 24 hpi.

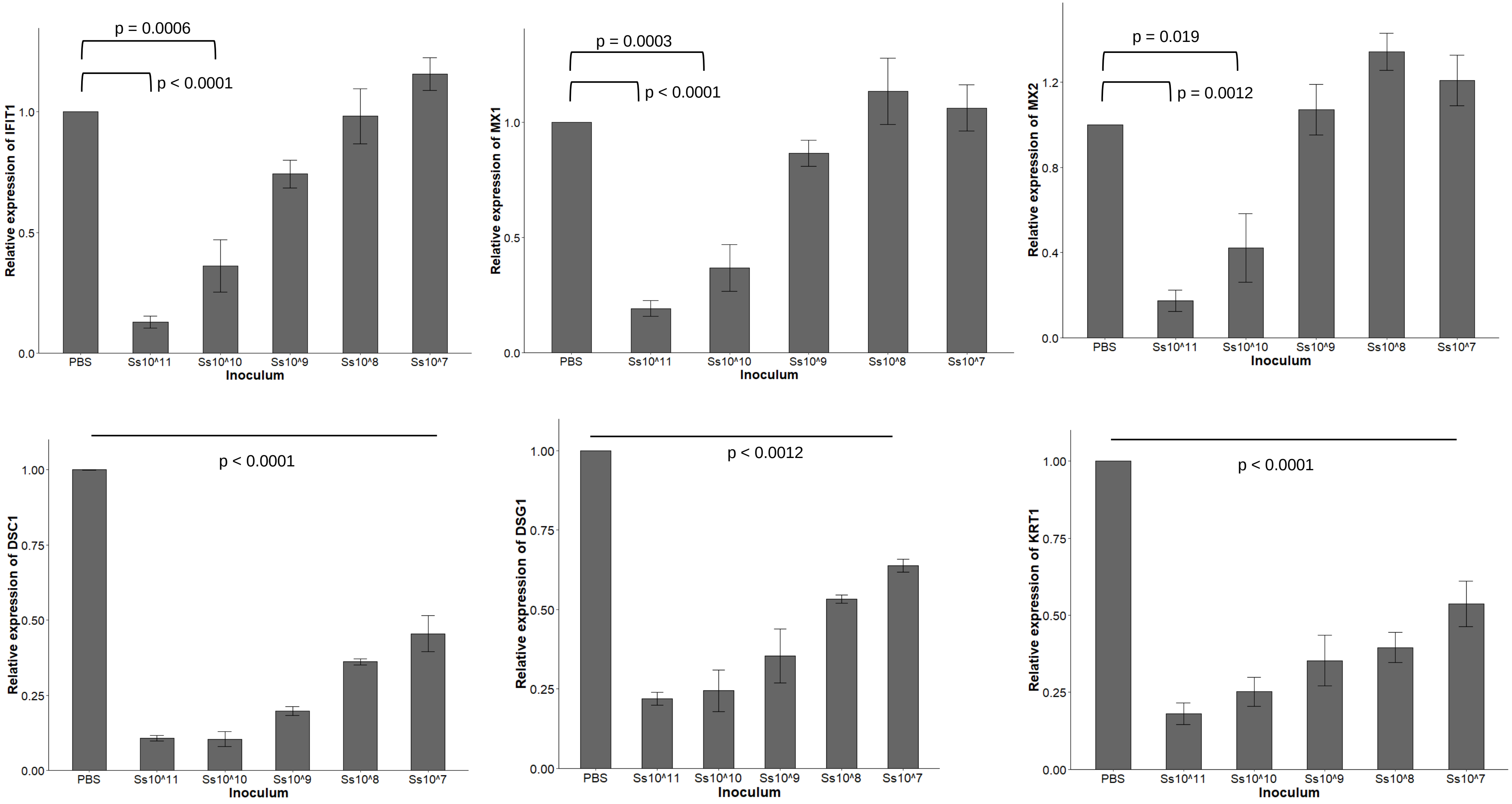

Fig. S3: Expression of significantly differentially regulated genes from RNAseq dataset measured by qRT-PCR in TIGK cells inoculated by a concentration range of SK36 EMVs (10^{11}-10^{7} particles/sample, 24 hpi). GAPDH was used as an internal control, values are relative to PBS-inoculated control set at 1. Bars represent averages of three independent biological replicates with standard error. P-values denote significance from PBS-inoculated control.

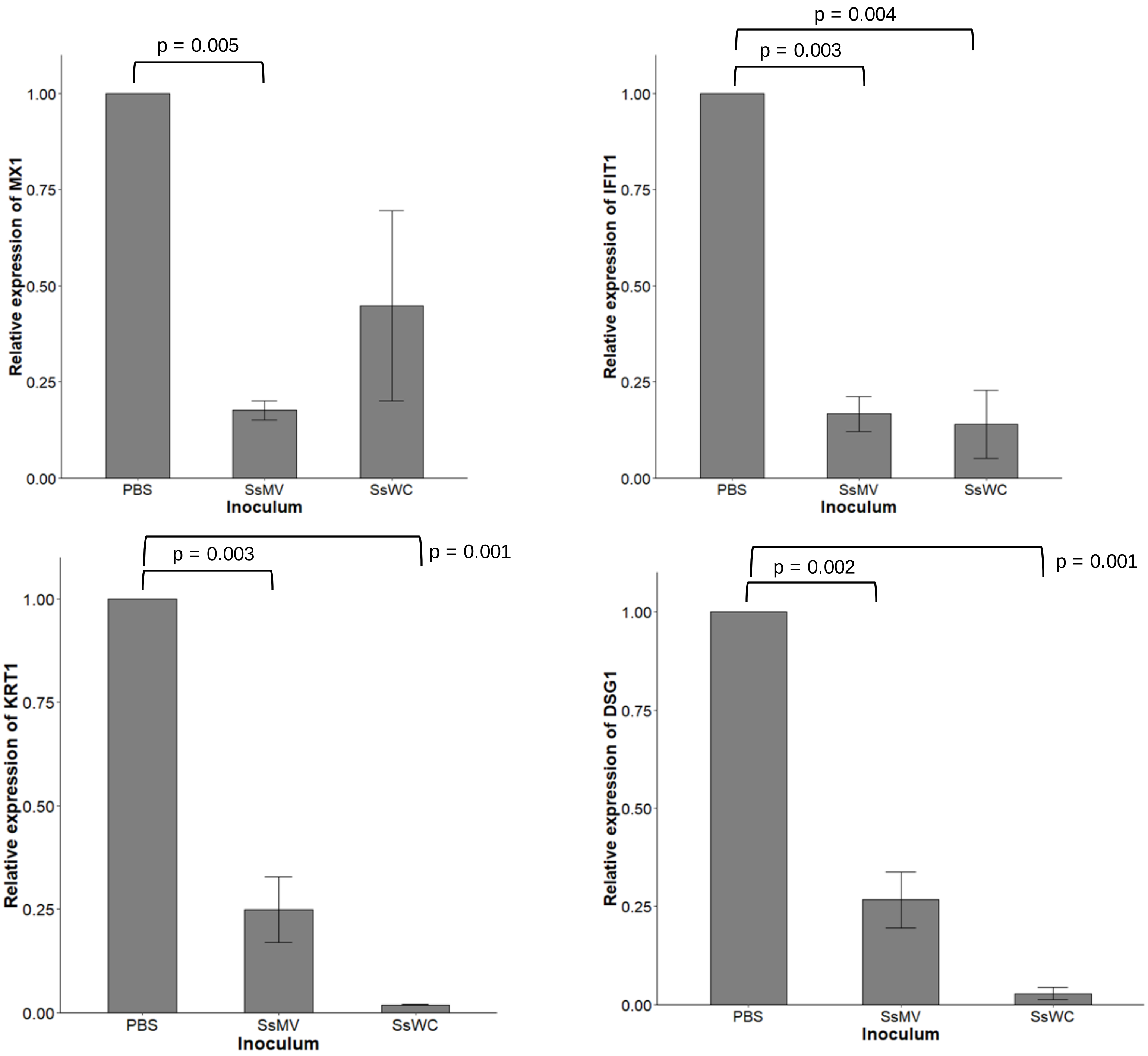

Fig. S4: Expression of significantly differentially regulated genes from RNAseq dataset measured by qRT-PCR in TIGK cells inoculated with SK36 EMVs (1011 particles/sample, denoted SsMV), SK36 whole cells (100MOI, denoted SsWC) or PBS and sampled at 24hpi. GAPDH was used as an internal control, values are relative to PBS-inoculated control set at 1. Bars represent averages of three independent biological replicates with standard error. P-values denote significance from PBS-inoculated control.
