# Supplementary material for: Immunostimulatory effects of Streptococcus sanguinis extracellular membrane vesicles protect oral gingival epithelial cells from periodontal pathobiont damage
Source: Infect Immun. 2025 Feb 19;93(3):e00535-24. doi: 10.1128/iai.00535-24 (PMC11895462; doi:10.1128/iai.00535-24)
Supplement: Supplemental material — Supplemental table legends. [file iai.00535-24-s0002.docx]

**Supplemental Tables legends**

**Supplemental Table 1)** General statistics of RNAseq dataset from TIGK cells inoculated with SK36 EMVs, 24hpi.

**Supplemental Table 2)** List of all significantly differentially regulated genes and DEseq2 output (P_adj_ < 0.05) from RNAseq dataset from TIGK cells inoculated with SK36 EMVs, 24 hpi.

**Supplemental Table 3)** List and sequences of all primers used in this study
